# Paralogues From the Expanded Tlr11 Gene Family in Mudskipper (*Boleophthalmus pectinirostris*) Are Under Positive Selection and Respond Differently to LPS/Poly(I:C) Challenge

**DOI:** 10.3389/fimmu.2019.00343

**Published:** 2019-02-28

**Authors:** Heng Tong Qiu, Jorge M. O. Fernandes, Wan Shu Hong, Hai Xu Wu, Yu Ting Zhang, Sheng Huang, Dong Teng Liu, Hui Yu, Qiong Wang, Xin Xin You, Shi Xi Chen

**Affiliations:** ^1^State Key Laboratory of Marine Environmental Science, College of Ocean and Earth Sciences, Xiamen University, Xiamen, China; ^2^Faculty of Biosciences and Aquaculture, Nord University, Bodø, Norway; ^3^Fujian Collaborative Innovation Center for Exploitation and Utilization of Marine Biological Resources, Xiamen, China; ^4^Shenzhen Key Laboratory of Marine Genomics, Marine and Fisheries Institute, BGI-Shenzhen, Shenzhen, China; ^5^State-Province Joint Engineering Laboratory of Marine Bioproducts and Technology, Xiamen University, Xiamen, China

**Keywords:** *Boleophthalmus pectinirostris*, TLR21, TLR22, TLR23, innate immunity, positive selection, LPS, poly(I:C)

## Abstract

Toll-like receptors (TLRs) are major molecular pattern recognition receptors, which are essential for triggering a series of innate immune responses against invading pathogens by recognizing their evolutionary conserved molecular patterns. The mudskipper, *Boleophthalmus pectinirostris* is exceptional among fishes due to its amphibious lifestyle and adaptation to living on mudflats. The whole-genome sequencing of *B. pectinirostris* has revealed that this species possesses an expansion of Tlr11 family [12 Tlr11 family genes (one *tlr21*, 4 *tlr22*, and 7 *tlr23*)] that we focused on in the present study. The full-length cDNA sequences of the 12 *tlrs* in *B. pectinirostris* were cloned and their deduced amino acid sequences possessed a typical TLR domain arrangement. Likelihood tests of selection revealed that these 12 Tlr11 family genes are under diversifying selection. A total of 13 sites were found to be positively selected by more than one evolution model, of which 11 were located in the ligand-binding ectodomain. The observed non-synonymous substitutions may have functional implications in antigen and pathogen recognition specificity. These 12 *tlrs* were highly expressed in immune-related tissues, i.e. spleen and kidney. *Tlr21* and *tlr22b* transcripts were significantly up-regulated by LPS, whereas *tlr22a, tlr22d, tlr23b, tlr23e, tlr23g* were significantly up-regulated by poly(I:C) in the spleen or/and kidney, which implies that the expanded Tlr11 family genes may play roles in protecting the fish from the invasion of gram-negative bacteria and double-stranded RNA viruses. The results from the present study suggested that the expansion of Tlr11 family genes in *B. pectinirostris* may recognize ligands from various pathogens found in the intertidal zone.

## Introduction

The Toll-like receptor (TLR) gene family is a class of pathogen recognition receptors (PRRs) that play crucial roles in the innate immune system by recognizing pathogen-associated molecular patterns (PAMPs) derived from various microbes (1, 2). TLRs interact with PAMPs from pathogens via their clusters of extracellular LRRs (leucine-rich repeats), resulting in conformational changes of TLRs. This further activates cytoplasmic Toll-Interleukin-1 receptor (TIR) domain to recruit cytosolic adaptor proteins, such as myeloid differentiation factor 88 (MyD88), and finally induces the production of multiple cytokines (3). Since the discovery of Toll in fruit fly (*Drosophila melanogaster*) in 1985 (4, 5), at least 28 TLRs have been identified in vertebrates and can be divided into six major families: TLR1 (TLRs 1, 2, 6, 10, 14, 15, 16, 18, 24, 25, 27 and 28), TLR3 (TLR3), TLR4 (TLR4), TLR5 (TLR5), TLR7 (TLRs 7, 8, 9), and TLR11 (TLRs 11, 12, 13, 19, 20, 21, 22, 23, and 26) (6). Bony fish are thought to have an ancient immune system and there is great scientific interest in comparing their innate and adaptive defense mechanism with mammals (7). So far, at least 21 Tlrs have been identified in fishes (8). TLR4 gene has been lost from the genomes of most fishes (9). TLR6 and TLR10 are absent in fishes (10). TLR5s, TLR14, TLR18 to TLR28 are considered to be termed as “teleost-specific TLRs” (8, 11).

Key features of the fish TLRs and the factors involved in their signaling cascade have high structural similarity to the mammalian TLR system. However, fish TLRs also exhibit very distinct features and large diversity which is likely derived from their diverse evolutionary history and the distinct environments that they occupy (7). In particular, some TLR genes in teleosts are known to be shaped by positive (diversifying or adaptive) selection, which enables them to cope with a large number of rapidly evolving pathogens (12–18).

The mudskipper *Boleophthalmus pectinirostris* (Linnaeus 1758) is a burrow-dwelling fish, widely distributing throughout the intertidal regions of China, Korea and Japan (19). *B. pectinirostris* is usually found on the soft mudflats of estuaries and coastal waters when they are exposed at ebb tide. Their behaviors, physiological and morphological features have been specialized and adapted for an amphibious lifestyle (20–25). Pathogenic bacteria adhere to and colonize mucosal surfaces of the susceptible host (26), or invade the body mainly through the skin, gill, or gut (27). The peculiar environment of the mudflats, which changes between flood and ebb tides, suggests that *B. pectinirostris* may have evolved specific immunity genes to adapt to their habitat. Interestingly, genomic study of *B. pectinirostris* showed that the fish species possesses the largest number (11 copies) of TLR13 in vertebrates sequenced so far (28). However, in the present study, based on the sequences of 11 Tlr13 of *B. pectinirostris*, we further cloned one more Tlr13 gene from *B. pectinirostris*. However, the phylogenetic analysis indicated that the 12 Tlr13 from *B. pectinirostris* should be classified as Tlr21, Tlr22, and Tlr23, which belong to TLR11 family. Similar TLR11 family expansion was also reported in Atlantic cod (*Gadus morhua*), and 12 *tlr22* paralogues of Atlantic cod responded differently to pathogenic challenge, which indicated that they are undergoing neofunctionalization via positive selection and can recognize bacterial pathogen-associated molecular patterns (13).

The aim of this study was to investigate if expansion of Tlr11 family in *B. pectinirostris* have been retained through adaptive evolution in order to provide special immune defense against pathogens from *Vibrio, Klebsiella, Salmonella*, etc. in intertidal mudflat (29, 30). After obtaining the full-length cDNA sequence of 12 Tlr11 family genes in *B. pectinirostris*, we conducted synteny analysis and chromosome localization. In order to assess the adaptive evolution of Tlr11 family genes of *B. pectinirostris*, positive selection analysis was performed. We further examined the tissue distribution and the expression profiles of these genes in response to lipopolysaccharide (LPS) and polyinosinic-polycytidilic acid [poly(I:C)] challenges.

## Materials and Methods

### Experimental Fish and Sampling

Adult mudskipper *B. pectinirostris* (body length 105–145 mm, body weight 20–45 g) used in this study were purchased from a seafood market in Xiamen, Fujian province, China. The fish were maintained in plastic tanks with 1.5 cm deep seawater at water temperature of 28–28.5°C, and salinity of 15 ppt. Before sampling, the fish were anesthetized with 0.01% MS222 (Sigma-Aldrich, St. Louis, MO, US). All experiment protocols were approved by the Institute of Animal Care and Use Committee of Xiamen University.

### cDNA Cloning, Gene Structures of *tlr21, tlr22*, and *tlr23* Paralogues

The fasta format of whole genome shotgun sequences of *B. pectinirostris* was downloaded from NCBI, and a local blast database was created with BioEdit (31). The partial fragments of previous published 11 *tlr13* genes of *B. pectinirostris* (28) were obtained from BGI (The Beijing Genomics Institute, Shenzhen), these sequences were used to search for the reference sequences from the database by BioEdit software. We found a new TLR11 family gene from local blast database based on the conserved cDNA sequence of TIR domain of *B. pectinirostris*. The start and stop codons of these genes were predicted by BLASTP searches (NCBI). Finally, we got sequences of 12 Tlr11 family genes in *B. pectinirostris*. Specific primers were designed to amplify the open reading frames of these genes (Supplementary Table 1). A cDNA library from kidney tissue was synthesized using the ReverAid First Strand cDNA Synthesis Kit (Thermo Scientific, USA) following the manufacturer's instructions and used to amplify these 12 *tlr* transcripts. Thirty-five cycles of standard PCR were performed on a Bio-Rad T100 Thermal Cycler, the annealing temperature was 58°C and the elongation time depended on the length of fragments amplified. No more than twenty cycles of nested PCR amplification with an annealing temperature of 58°C were performed when necessary.

The full-length end cDNA sequences of these 12 *tlr* genes were obtained using a SMART RACE cDNA Amplification kit (BD, Clontech) following the manufacturer's instructions, and the combined PCR sequences were used to deduce the full-length cDNA sequences of the 12 *tlr* genes. Briefly, total RNA extracted from the fresh kidney was used to synthesize the RACE Ready first-strand cDNA. The 3′RACE cDNA was synthesized using 3′-CDS Primer A, and the 5′ RACE cDNA was synthesized using 5′-CDS Primer A and SMARTer IIA oligo. RACE primers for these 12 genes were designed based on the sequence information of the fragments obtained above (Supplementary Table 2). The PCR products were cloned into pMD19-T simple vector (TaKaRa Dalian, China) and sequenced by Invitrogen Ltd. (Guangzhou, China). Intron-exon boundaries of these 12 *tlrs* were identified using corresponding genome sequences and gene structure display server (http://gsds.cbi.pku.edu.cn/). Furthermore, we searched for the highly conserved tandem repeat sequences in the full-length cDNA sequences of these 12 *tlrs* from *B. pectinirostris* using the online software “Tandem Repeats Finder” (32).

### Synteny Analysis and Chromosome Location of *tlr21, tlr22*, and *tlr23* Paralogues

Synteny analysis was performed manually based on the genome assemblies of large yellow croaker (*Larimichthys crocea*) (genome assembly accession no. GCF_000972845.1), green-spotted pufferfish (*Tetraodon nigroviridis*) (GCA_000180735.1), tiger pufferfish (*Takifugu rubripes*) (GCF_000180615.1), yellowtail kingfish (*Seriola lalandei dorsalis*) (GCA_002814215.1), amberjack (*Seriola dumerili*) (GCF_002260705.1), Asian seabass (*Lates calcarifer*) (GCF_001640805.1).

A high-quality chromosome map comprising 916.23 Mb (93.2%) of *B. pectinirostris* entire sequence was constructed as part of our program, and will be published separately (data not shown). The full-length cDNA sequences of these 12 *tlr* genes were used to determine their locations in the 23 pseudo-chromosomes of *B. pectinirostris* by BLAST searches (https://blast.ncbi.nlm.nih.gov/Blast.cgi).

### Phylogenetic Analyses

The deduced amino acid sequences of the expanded Tlr11 family genes were obtained using the ExPASy Translate Tool (http://www.expasy.ch/tools/dna.html). Protein domains, signal peptide, and transmembrane regions were predicted using SMART (http://smart.embl-heidelberg.de/), SignalP 4.1 (http://www.cbs.dtu.dk/services/SignalP/) and the TMHMM Server v. 2.0 (http://www.cbs.dtu.dk/services/TMHMM/), respectively. A homology search was performed using the BLAST tool at NCBI (http://www.ncbi.nlm.nih.gov/BLAST/). The phylogenetic reconstruction was performed using MEGA software 7 (33) by the Neighbor-joining method, and a bootstrap consensus tree was inferred from 1,000 replicates. We also constructed a maximum likelihood phylogenetic tree in MEGA software 7 (33) using the Tamura 3-parameter model and γ distributed rates with invariant sites (G+I) and 5 γ categories, and a bootstrap consensus tree was inferred from 1,000 replicates. GenBank accession numbers of *tlr* genes for alignment of amino acids and phylogenetic tree construction are as follows: *Anser cygnoides* TLR21 (AMB20882); *Gallus gallus* TLR21 (NP_001025729); *Epinephelus coioides* TLR21 (AEK49148); *Takifugu rubripes* TLR21 (AAW69371); *Oreochromis niloticus* TLR21 (AHK13949.1); *Gadus morhua* TLR21 (AFK76484.1); *Salmo salar* TLR21 (CDH93614.1); *Danio rerio* TLR21 (CAQ13807); *Anolis carolinensis* TLR21 (XP_008123135.2); *Xenopus tropicalis* TLR21 (XP_002936443.2); *Epinephelus coioides* TLR22 (AGA84053.1); *Scophthalmus maximus* TLR22 (AIC75881.1); *Takifugu rubripes* TLR22 (AAW69372.1); *Larimichthys crocea* TLR22 (XP_010741403); *Tetraodon nigroviridis* TLR22 (CAG05452.1); *Gadus morhua* TLR22b (AFK76486.1); *Gadus morhua* TLR22d (AFK76488.1); *Gadus morhua* TLR22g (AFK76491.1); *Gadus morhua* TLR22i (AFK76493.1); *Miichthys miiuy* TLR23 (ALJ55575.1); *Takifugu rubripes* TLR23 (AAW70378.1); *Gadus morhua* TLR23a (AFK76497.1); *Gadus morhua* TLR23b (AFK76498.1); *Labrus bergylta* TLR23 (XP_020513361.1); *Tetraodon nigroviridis* TLR23 (CAF93842.1); *Seriola dumerili* TLR23a (XP_022616855.1); *Seriola dumerili* TLR23b (XP_022603128.1); *Seriola dumerili* TLR23c (XP_022603127.1); *Lates calcarifer* TLR23a (XP_018537426.1); *Lates calcarifer* TLR23b (XP_018546010.1); *Lates calcarifer* TLR23c (XP_018517760.1); *Seriola lalandi dorsalis* TLR23a (XP_023286622.1); *Seriola lalandi dorsalis* TLR23b (XP_023252716.1); *Seriola lalandi dorsalis* TLR23c (XP_023252714.1).

### Analyses of Positive Selection

The complete coding sequences (CDS) of the 12 Tlr11 family genes from *B. pectinirostris* (Table 1) were first aligned with MUSCLE (www.ebi.ac.uk/Tools/msa/muscle) and a codon alignment was obtained using RevTrans 2.0b (www.cbs.dtu.dk/services/RevTrans-2.0/web) followed by Codon Align (www.hiv.lanl.gov). The N- and C- terminal portions (60 and 12 codons, respectively) of the codon aligned TLR11 sequences were too variable and hence not included in the following analysis. Gaps present in more than one sequence were also manually removed. The refined codon alignment used in the selection tests comprised 93% of the total CDS and did not have any stop codons. This alignment was used to construct a maximum likelihood phylogenetic tree in MEGA7 (33) using the Tamura 3-parameter model and γ distributed rates with invariant sites (G+I) and 5 γ categories. A bootstrap consensus tree was inferred from 1,000 replicates.

**Table 1:** The characterization of *tlr21, tlr22*, and *tlr23* paralogues in *B. pectinirostris*.

**Gene name**	**Full cDNA (bp)**	**ORF (bp)**	**5′UTR (bp)**	**3′UTR (bp)**	**No. of exons**	**Number of amino acids**	**Scaffold**	**Genbank accession no**.
*tlr21*	3520	2898	255	367	1	965	scaffold9	MH744540
*tlr22a*	3820	2853	72	895	4	950	scaffold291	MH744541
*tlr22b*	3559	2697	165	697	4	898	scaffold890	MH744542
*tlr22c*	3219	2877	74	268	4	958	scaffold183	MH744543
*tlr22d*	4637	2856	1524	257	3	951	scaffold103	MH744544
*tlr23a*	3694	2814	194	686	5	937	scaffold936	MH744545
*tlr23b*	2878	2748	45	85	4	915	scaffold1155	MH744546
*tlr23c*	4825	2883	37	1905	5	960	scaffold294	MH744547
*tlr23d*	4904	2820	436	1648	4	939	scaffold1045	MH744548
*tlr23e*	4410	2766	1410	234	3	921	scaffold219	MH744549
*tlr23f*	3451	2805	37	609	5	934	scaffold294	MH744550
*tlr23g*	3574	2835	9	730	5	944	scaffold50	MH744551

The average number of synonymous and non-synonymous (amino acid-changing) substitutions, insertions and deletions in the codon alignments were calculated using SNAP (www.hcv.lanl.gov). This algorithm performs pairwise comparisons between all sequences in the alignment using the method developed by Nei and Gojobori (34). In protein coding genes, the ratio (ω) between non-synonymous (dN) and synonymous (dS) substitution rates is related to evolutionary constraints at the protein level (35). A value of ω>1 indicates positive Darwinian selection, whereas ω < 1 suggests negative or purifying selection. A codon based Z-test of selection was performed to test the hypothesis of positive selection in MEGA7 (33) using the modified Nei-Gojobori method with Jukes-Cantor correction (36). The hypothesis of positive selection was further tested using the likelihood methods implemented in the CODEML program of PAML v4.9 (37) and the Datamonkey adaptive evolution server (38), as detailed elsewhere (39). In PAML, the data set was fitted to 6 models of codon substitution: M0 (one ratio), M1 (two site classes), M2 (positive selection with three site classes, M3 (discrete), M7 (β) and M8 (continuous). Bayesian posterior probabilities were calculated for positively selected sites using naïve empirical Bayes in the case of model M3 or Bayes empirical Bayes for models M2 and M8. Likelihood ratio tests were used to compare the corresponding models with and without selection (i.e., M2 vs. M1, M3 vs. M0, and M8 vs. M7). FEL, SLAC and REL analyses were performed in Datamonkey to calculate dN-dS values for each codon, along with the corresponding probability values. Overall differences in diversifying selection between paralogous genes were determined with GA-branch method implemented in Datamonkey.

The three-dimensional structure of *B. pectinirostris* Tlr23a was predicted by SWISS-MODEL (40). In brief, structural template searches against the SWISS-MODEL template library were performed with BLAST and HHBlit. The highest quality template was then selected for model building based on the target-template alignment using ProMod3. The global and per-residue model quality has been assessed using the QMEAN scoring function (41). For improved performance, weights of the individual QMEAN terms have been trained specifically for SWISS-MODEL. The local quality plot was shown in Supplementary Figure 1. Positively selected codons were identified in the three-dimensional protein using the web-based viewer iCn3D at NCBI (www.ncbi.nlm.nih.gov/Structure/icn3d/full.html).

### Expression of *tlr21, tlr22*, and *tlr23* Paralogues in Different Tissues

To further explore the potential functions of *tlr21, tlr22*, and *tlr23* paralogues in *B. pectinirostris*, the basal expression levels of these 12 Tlr11 family genes in different tissues were quantified by real-time qPCR. Tissues including brain, heart, spleen, gills, liver, intestine, testis, seminal vesicle, ovary, skin, eye, kidney, blood cells were collected separately from seven *B. pectinirostris*. All the samples were snap-frozen in liquid nitrogen and stored at −80°C until analyses. Total RNA extraction, cDNA synthesis and real-time qPCR were performed as described in section Real-Time qPCR.

### Expression of *tlr21, tlr22*, and *tlr23* Paralogues in Response to LPS and Poly(I:C) Challenges

LPS is the main component of the cell surface of Gram-negative bacteria and poly(I:C) is used here as a model of double-stranded RNA virus infection. To investigate the potential functions of the 12 Tlr11 family genes in *B. pectinirostris*, the expression levels of these genes in the spleen and kidney were analyzed following intraperitoneal injections of LPS and poly(I:C). Male *B. pectinirostris* with similar size (body length 119–132 mm, body weight 29.5–33.6 g) were transported live in plastic tanks and acclimated to laboratory conditions (seawater at salinity 15 ppt and temperature 28–28.5°C) for 1 day. For the LPS challenge experiment, fish were randomly divided into two groups and each fish was intraperitoneally injected with LPS (Sigma, *E. coli* 0127:B8) dissolved in sterile 100 μL PBS at the dose of 0.1 mg in the treated group or with 100 μL sterile PBS in the control. At 3, 6, 12, 24 h post injection (hpi), the spleen and kidney of five or six individuals from each group at each time point were surgically sampled, frozen immediately in liquid nitrogen and stored at −80°C until analyses. For the poly(I:C) challenge experiment, the fish were prepared as described above. Each fish was intraperitoneally injected with poly(I:C) (Sigma, P0913) dissolved in sterile 100 μL PBS at the dose of 0.1 mg in the treated group or with 100 μL sterile PBS in the control group. The spleen and kidney of five or six individuals from each group at 3, 6, 12, and 24 hpi were surgically collected. Total RNA extraction and cDNA synthesis of these organs were conducted as described in section Real-Time qPCR. Real-Time qPCR was performed as described in section Real-Time qPCR.

### Real-Time qPCR

Total RNA was extracted from tissues using the RNAiso Plus (TaKaRa Dalian, China) and treated with RNase-free DNase I (Fermentas, USA) to eliminate contaminated genomic DNA. 1.5 μg total RNA was used for the synthesis of the first strand cDNAs using the RevertAid first stand cDNA synthesis kit (Thermo Scientific, USA). The gene specific primers used for real-time qPCR analysis and amplicon lengths are listed in Table 2. Amplification was conducted on a qTOWER 2.2 Real-Time PCR (Analytik Jena AG, Jena, Germany) using the PowerUp SYBR Green Real-time PCR Master Mix kit (Thermo Scientific, USA). Each 20 μL reaction contained 10 μL of PowerUp SYBR Green Real-time PCR Master Mix, 2 μL of cDNA template, 1 μL of each primer (10 μM), and 6 μL of water. Sterilized water was substituted for the cDNA in negative control samples. The amplification program was performed as follows: predenaturation at 95°C for 2 min followed by 40 cycles at 95°C for 15 s, 60°C for 30 s, and 72°C for 30 s. Each sample was analyzed in duplicate.

**Table 2:** The primers for Real-time qPCR in this study.

**Primer**	**Sequence (5′−3′)**	**Amplicon length (bp)**
*tlr21* rtF	AACTCTGTCTACATCACAGAGAC	272
*tlr21* rtR	CAGATAGGTCTTCTTAAGCATGAC	
*tlr22a* rtF	CTGGAGAACGATCAAGGCTGGAAG	146
*tlr22a* rtR	CACTCGCTCTGTAGATATCGTCTG	
*tlr22b* rtF	TTCAGCAGATTTCACCTGAGTTAC	241
*tlr22b* rtR	CTTCAGCGATGTTCTCCACGATG	
*tlr22c* rtF	TATAGAGAACTAGTGCCACATCTG	214
*tlr22c* rtR	GCTCATCGAACAAACGGAAACTG	
*tlr22d* rtF	GCGTAGAGGATCAGTACGATG	178
*tlr22d* rtR	CTTCCATAAATGGCATCAGCAATG	
*tlr23a* rtF	GCTGGAGGCTTTGTCTGCACCAC	186
*tlr23a* rtR	CGTCTTTGTGCTCATCGAACAGAC	
*tlr23b* rtF	CGTTCGTTTCCTACAACGTTCACG	263
*tlr23b* rtR	CACGTCCTTCTGCTCATCGAACAG	
*tlr23c* rtF	ATTTACCTACCTTTTATACCCTAC	321
*tlr23c* rtR	GCTCAAGTACAAATAGAGTCAATC	
*tlr23d* rtF	GTTTCTAAGGACAAAGCTCATGAC	247
*tlr23d* rtR	CACAGACTTATTTTGGAGCATCTG	
*tlr23e* rtF	TGTGTCCTACAACTGTCACGATG	167
*tlr23e* rtR	ACAGAGTCTTCCTGCTTCTGTAG	
*tlr23f* rtF	AACAACAAAATTGATCATATTTCC	248
*tlr23f* rtR	CGAAATTTGGTCAAAGTATCTGAG	
*tlr23g* rtF	CTACAACGTTCATGATGAGAACTG	251
*tlr23g* rtR	CATCCTTCTGCTCATCAAACAGAC	
*eef1α* rtF	TGGAACCTCTCAGGCTGACT	275
*eef1α* rtR	ATCCAGAGATGGGCACAAAG	

*F, forward; R, reverse*.

### Statistical Analysis

Statistical analysis was performed using Graphpad software and the relative abundance of mRNA for target genes was calculated using 2^−ΔΔCt^method (42) with the eukaryotic translation elongation factor 1α (*eef1*α, Genbank accession No. XM_020932525.1) gene as the reference. Data were presented as mean ± standard error of the mean (SEM) and the Student's *t*-test was used to assess statistical differences of expression levels between groups. For multiple group comparison, one-way ANOVA followed by Tukey's test was used for statistical analysis. Differences were considered to be statistical significance when *p* < 0.05.

## Results

### cDNA Sequences of *tlr21, tlr22*, and *tlr23* Paralogues

**Figure 1:**
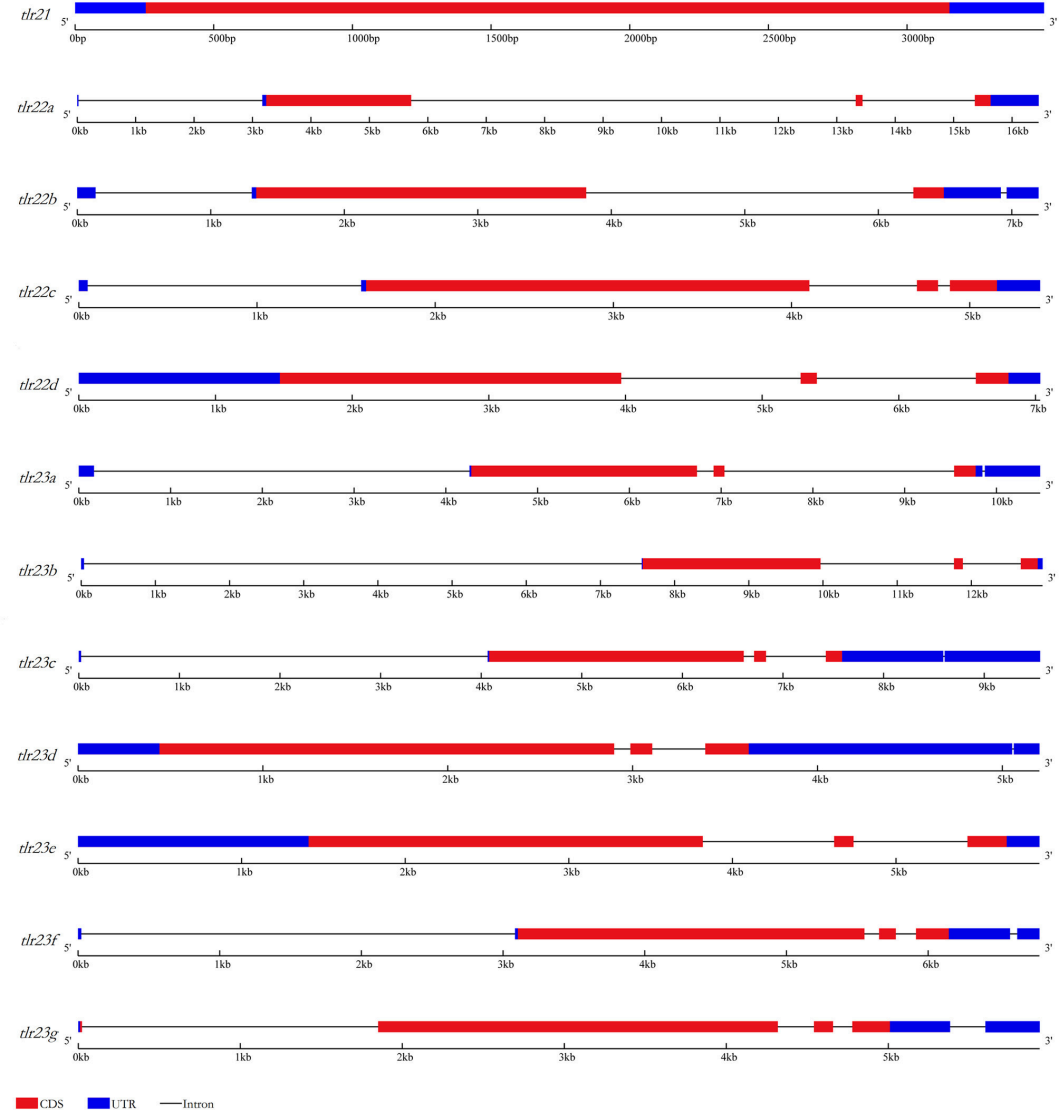
Gene structures of *tlr21, tlr22, tlr23* paralogues in *B. pectinirostris*. Graphical representation of *tlr21, tlr22*, and *tlr23* gene structures. UTRs and CDS are represented in light blue and red, respectively. Introns are indicated by continuous lines.

The characterization of *tlr21, tlr22*, and *tlr23* paralogues is summarized in Table 1 and their structures are showed in Figure 1, based on the genome assembly of *B. pectinirostris*. Although the lengths of these 12 *tlr* cDNAs varied from 2,878 to 4,904 bp, the deduced protein sizes were conserved, ranging from 898 to 965 amino acids. With 3,520 bp, the *tlr21* full cDNA was encoded by a single exon, including 255 bp 5′-UTR, 367 bp 3′-UTR, and the 2,898 bp complete coding region corresponding to a 965 aa protein. Among *tlr22* and *tlr23* orthologs, only *tlr22d* and *tlr23e* comprised 3 exons and had longer 5′-UTRs, while *tlr23c* and *tlr23d* contained longer 3′-UTRs.

Several highly conserved tandem repeats were found mainly within 5′-UTR or 3′-UTR of *tlr22b, tlr23a, tlr23d* (Supplementary Table 3) and *tlr22d* (Supplementary Figure 2). The longest tandem repeats spanning 563 bp were identified in the 5′-UTR of *tlr22d*, and the shortest tandem repeats spanning 96 bp were found in the 5′-UTR of *tlr22b*. The copy number of 11 bp tandem repeat within the 3′-UTR of *tlr22b* was up to 50. The tandem repeats of *tlr23a* started at the 5′ end of the cDNA. The tandem repeats of *tlr23d* were completely distributed within 3′-UTR.

### Synteny Analysis and Chromosome Location of *tlr21, tlr22*, and *tlr23* Paralogues

**Figure 2:**
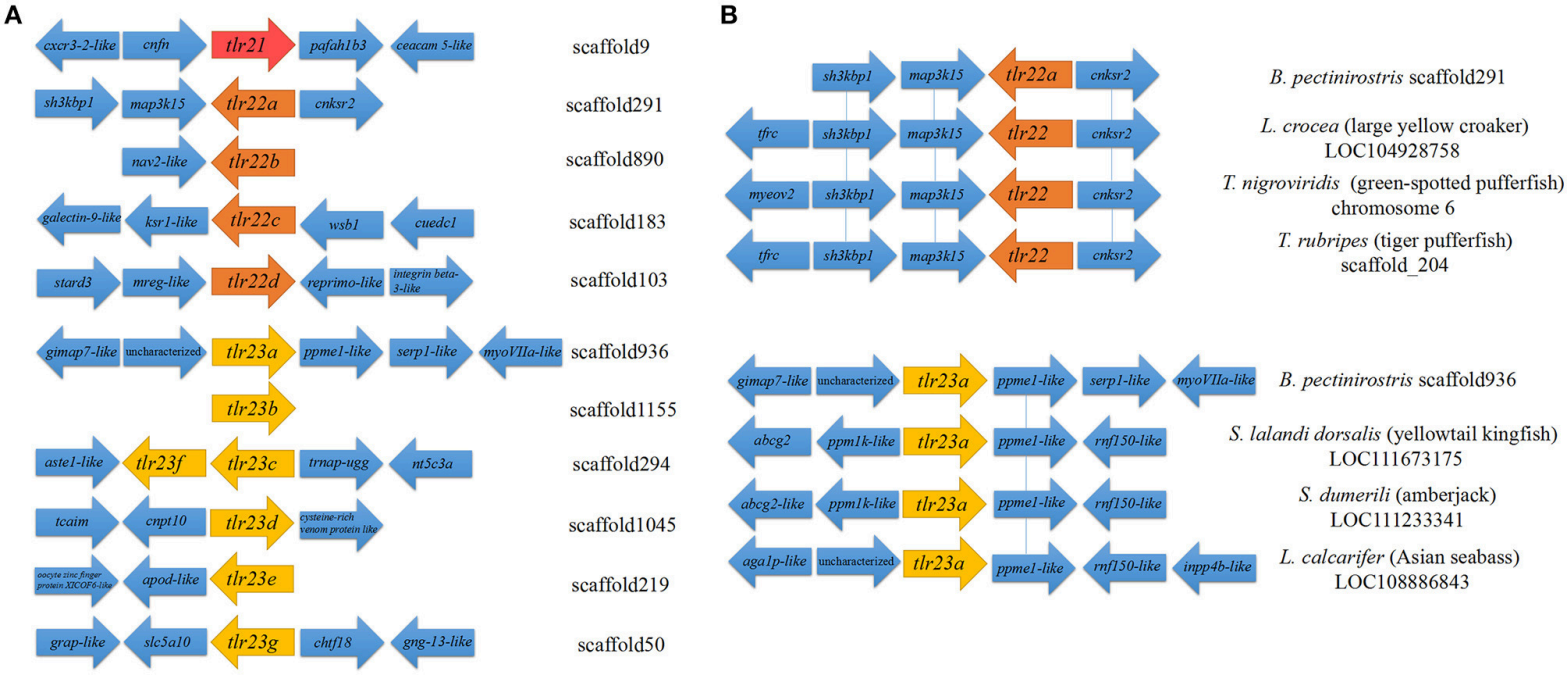
Partial synteny map of the genomic region surrounding Tlr11 family genes in *B. pectinirostris*. **(A)** Partial map of the genomic regions surrounding the *tlr21, tlr22*, and *tlr23* paralogues in *B. pectinirostris*. **(B)** Partial synteny map between *tlr22a, tlr23a* from *B. pectinirostris* and *tlr22, tlr23a* from large yellow croaker (*L. crocea*), green-spotted pufferfish (*T. nigroviridis*), tiger pufferfish (*T. rubripes*), yellowtail kingfish (*S. lalandi dorsalis*), amberjack (*S. dumerili*), and Asian seabass (*L. calcarifer*). The vicinity of *tlr22* and *tlr23* paralogues are connected by blue lines to show synteny amongst different species.

Both *tlr23c* and *tlr23f* were present in the same scaffold (scaffold294), while the other paralogues were mapped to different scaffolds (Table 1; Figure 2A). Partial synteny analysis based on the current mudskipper genome build revealed conservation between *tlr22a* in *B. pectinirostris* and *tlr22* coding genes in large yellow croaker, green-spotted pufferfish and tiger pufferfish, within the genomic region containing *sh3kbp1, map3k15*, and *cnksr2* (Figure 2B). *Tlr23a* in *B. pectinirostris* and *tlr23a*s in yellowtail kingfish, amberjack and Asian seabass were adjacent with the same gene “*ppme1-like*” (Figure 2B).

**Figure 3:**
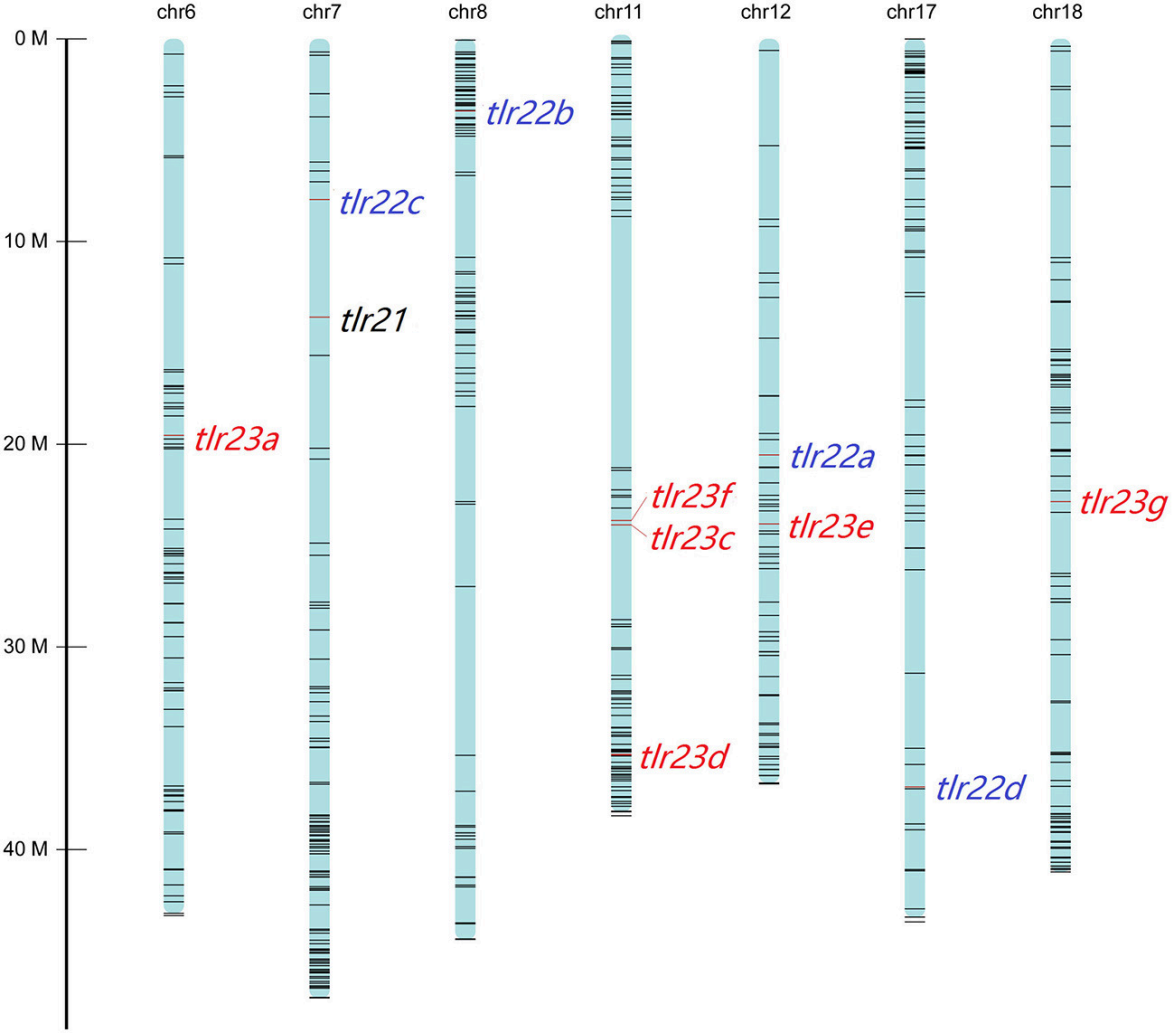
Distributions of *tlr21, tlr22, tlr23* paralogues in pseudo-chromosomes of *B. pectinirostris*. The genes were indicated with red lines. The SNP markers in each chromosome are showed in black lines.

All these 12 *tlr* genes, except *tlr23b*, mapped on seven chromosomes of *B. pectinirostris* (Figure 3, Supplementary Table 4). *Tlr21* was located at chr7, *tlr22a, tlr22b, tlr22c*, and *tlr22d* were located at chr12, chr8, chr7, and chr17, respectively. Among *tlr23* genes, three of them (*tlr23c, tlr23d, tlr23f*) were present in the same chromosomal region (chr11), while *tlr23a* and *tlr23g* were found in chr6 and chr18, respectively. The location of *tlr23b* needs to be further explored.

### Phylogenetic Analysis and Protein Domain Arrangements of *tlr21, tlr22*, and *tlr23*i Paralogues in *B. pectinirostris*

**Figure 4:**
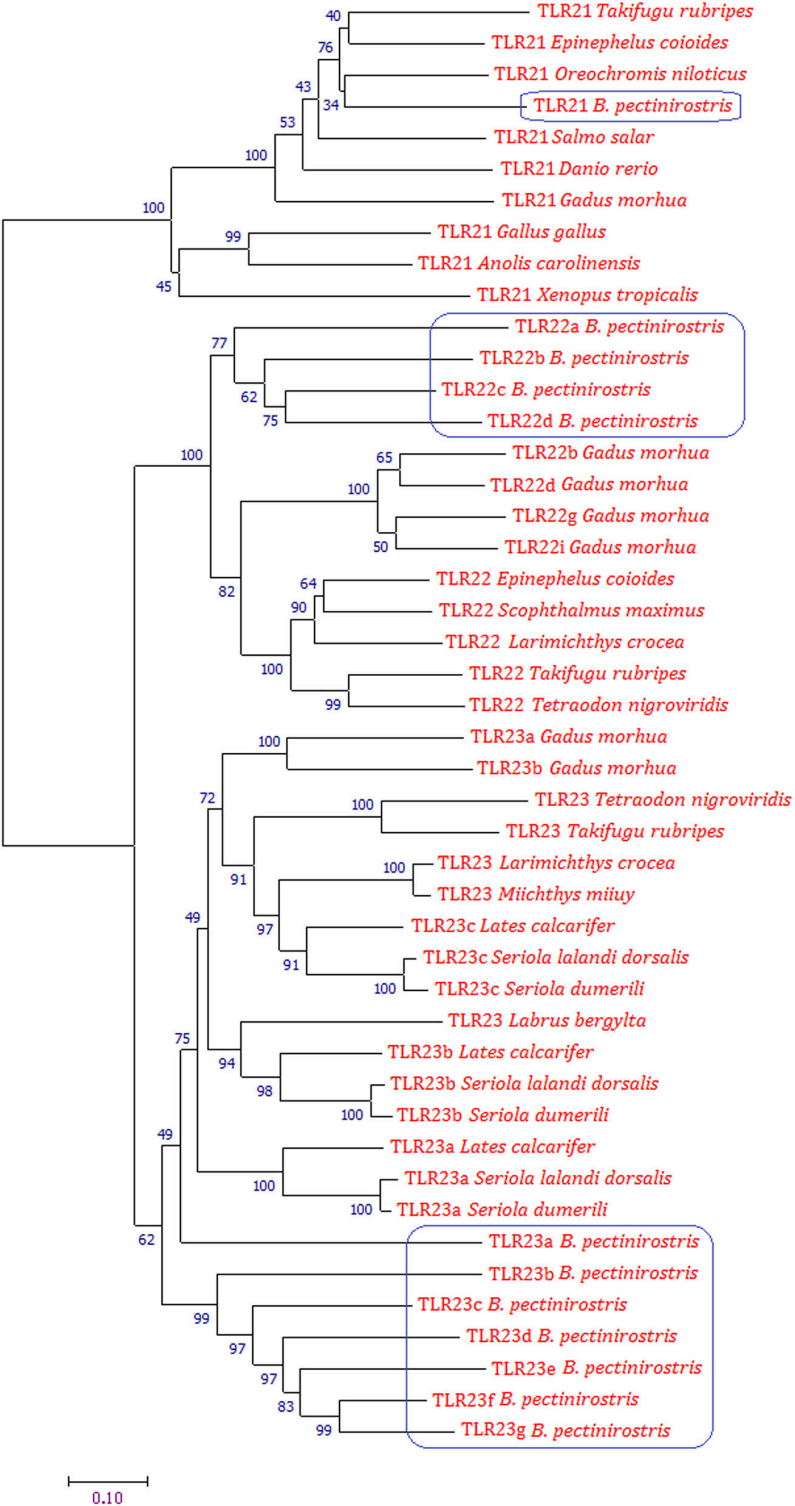
Phylogenetic analysis of TLR21, TLR22, and TLR23 using MEGA 7 by the Neighbor-joining method and 1,000 replications of bootstrap. Proteins of *B. pectinirostris* are highlighted within blue box.

The phylogenetic tree was constructed using the Neighbor-joining method on the basis of deduced amino acid sequences of TLR21, TLR22, and TLR23 of vertebrates (Figure 4). Phylogenetic analysis showed that TLR21, TLR22, and TLR23 constituted three major groups. In the tree, all TLR21 were grouped under a single clade, while TLR22 and TLR23 formed a separate cluster. The Tlr22 orthologs in *B. pectinirostris* were grouped together. All Tlr23 orthologs except Tlr23a clustered together in *B. pectinirostris*. Similar results were also found in the phylogenetic tree based on Maximum Likelihood method (Supplementary Figure 3).

**Figure 5:**
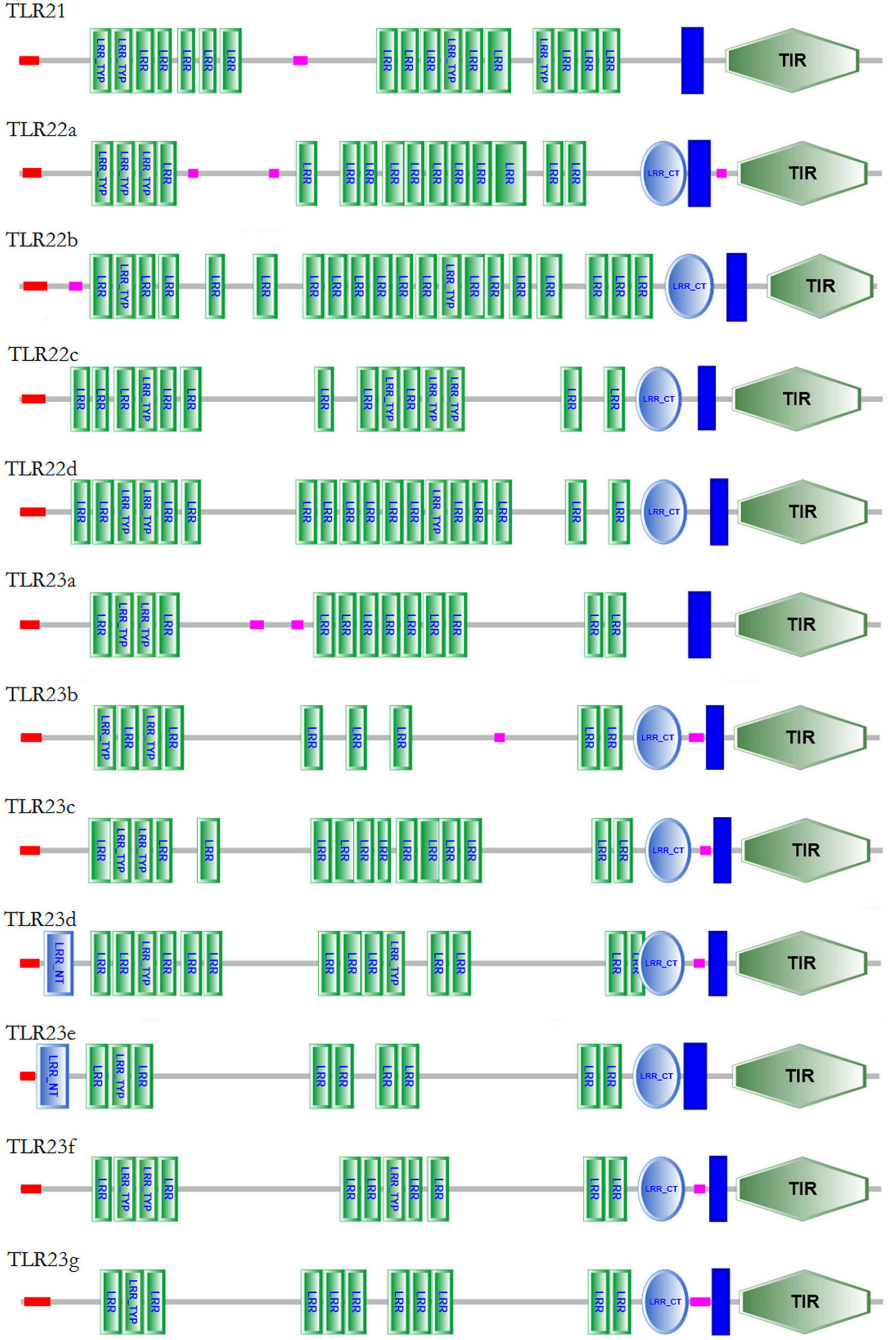
Protein domain structures of Tlr21, Tlr22, and Tlr23 proteins in *B. pectinirostris*. The domain organizations of Tlr21, Tlr22, and Tlr23 in *B. pectinirostris* were predicted using SMART, SignalP, and TMHMM analyses. LRR, leucine-rich repeat; LRR-TYP, leucine-rich repeat typical subfamily; TIR, Toll/IL-1 receptor; NT, N-(nitrogen) terminal; CT, C-(carboxyl) terminal; 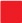, signal peptide; 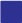, transmembrane region; 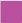, low complexity region.

The protein domain arrangements of *tlr21, tlr22*, and *tlr23* paralogues in *B. pectinirostris* are presented in Figure 5. All Tlrs amino acid sequences comprised of a signal peptide, several LRRs, a transmembrane domain and a TIR domain. These Tlrs contained various numbers of LRR domains, and the LRR number in each TLR ranged from 10 to 21: 17 (Tlr21), 16 (Tlr22a), 21 (Tlr22b), 15 (Tlr22c), 19 (Tlr22d), 13 (Tlr23a), 10 (Tlr23b), 16 (Tlr23c), 16 (Tlr23d), 11 (Tlr23e), 12 (Tlr23f), 12 (Tlr23g). Most of these Tlrs had C-terminus LRRs (LRR-CT) with the exception of Tlr21 and Tlr23a. Only Tlr23d and Tlr23e contained N-terminus LRRs (LRR-NT). Besides, Tlr22b contained a shorter TIR domain than other Tlrs.

### Molecular Evolution of the *Tlr11* Family in *B. pectinirostris*

**Figure 6:**
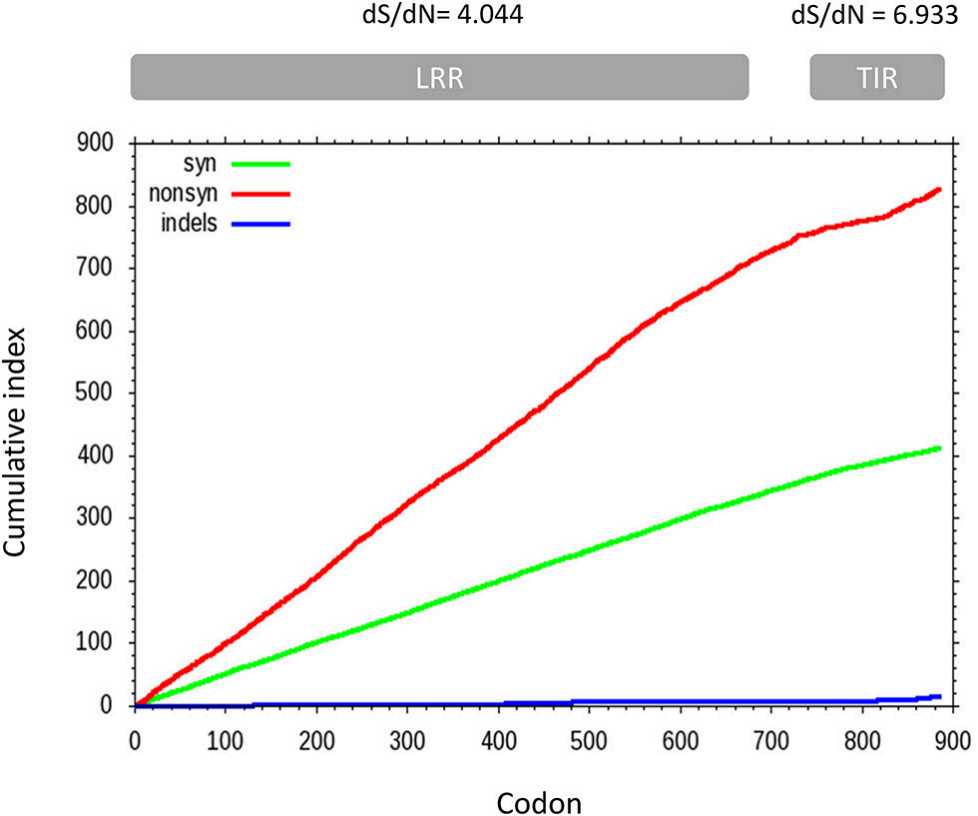
Cumulative non-synonymous (dN, red) and synonymous (dS, green) substitutions for all pairwise comparisons between 12 *tlr11* paralogues in *B. pectinirostris*. Insertions and deletions are shown in blue. The average dS and dN of all pairwise comparisons for the LRR and TIR domains are indicated above the corresponding regions. Divergence at non-synonymous sites is higher in the LRR region than in the TIR domain.

A sliding window analysis of the complete coding sequences of *tlr21, tlr22*, and *tlr23* paralogues revealed that the cumulative number of non-synonymous mutations per codon (dN) exceeded the number of synonymous substitutions (dS) and that their occurrence was not uniform throughout the *tlr21, tlr22*, and *tlr23* coding sequences (Figure 6). The average dS of all pairwise comparisons was higher than dN but different between the LRR and TIR domains, with dS/dN ratios of 4.044 and 6.933, respectively. A pairwise codon-based *Z*-test did not reject the null hypothesis of strict-neutrality (dN = dS) in favor of positive selection (dN > dS) (Supplementary Table 5). Nevertheless, the dN/dS ratios (ω) varied between *tlr11* genes and were highest in *tlr23* paralogues, particularly in *tlr23c-g* (Supplementary Figure 4).

**Figure 7:**
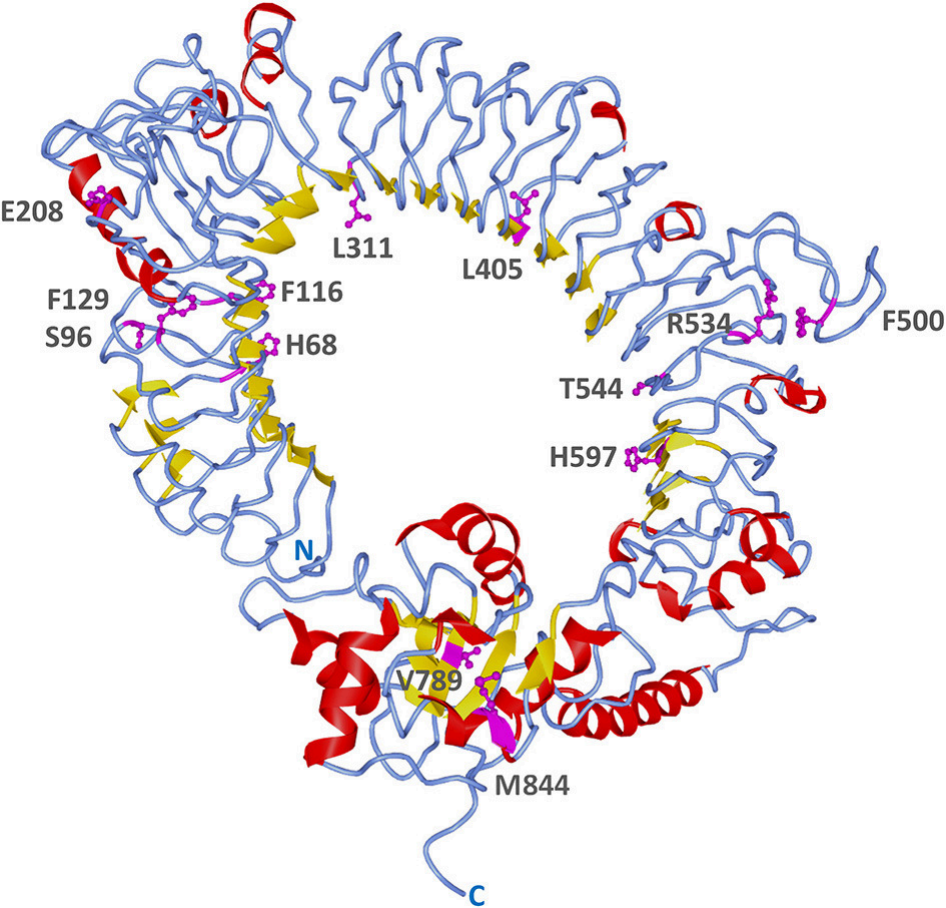
Positively selected sites on *B. pectinirostris* Tlr23a. Its tertiary structure was obtained by homology modeling using human TLR5 (PDB 3J0A) as template. Sites found to be under positive selection by more than one likelihood model are indicated in magenta and their side chains are shown. Residue numbers are based on the codon alignment used for positive selection analysis (Supplementary Figure 5).

A more detailed site-specific analysis was performed using likelihood models to identify codons under diversifying selection. Likelihood ratio tests in PAML showed that models allowing for positive selection fitted the data better than those that did not (M3 vs. M0, 2ΔLnL = 1614, *p* = 0; M2 vs. M1, 2ΔLnL = 45, *p* = 0; M8 vs. M7, 2ΔLnL = 40.6, *p* = 0) (Table 3). Models, M2, M3, and M8 identified 4, 46 and 3 codons under positive selection (Bayesian posterior *p* < 0.05) and ω values of 5.39, 1.02, and 2.8, respectively. The best model of nucleotide substitution was 012032 with an Akaike information criterion of 63195. FEL and SLAC analyses found 18 and 3 codons under positive selection with *p* < 0.1 and REL identified 6 positively selected sites with Bayes factor >50 (Table 3). In total, 13 codons were identified by more than one likelihood model as being under significant positive selection pressure. In particular, codon 68 (F, H, K, N, R, S, T, V, or W) was flagged by all models, except REL (Supplementary Figure 5). Only two out of these 13 codons were present within the TIR domain, whereas 11 were found in the ectodomain. Most positively selected sites (8 out of 13) were found in LRR regions, especially in the coils on the convex surface of this horseshoe-shaped domain. Two sites under diversifying selection (405 and 597) were located in beta sheets within the concave surface (Figure 7, Supplementary Figure 5).

**Table 3:** Positively selected sites in mudskipper *tlr21, tlr22, and tlr23* paralogues.

**Method**	**Model**	**Parameter estimates**	**Ln likelihood**	**Model comparison**	**Positively selected sites^a^**
CODEML^b^	M0: neutral	ω = 0.32	−30533.3		None
	M1: nearly neutral	ω_0_ = 0.15, ω_1_ = 1.00 *p*_0_ = 0.50, *p*_1_ = 0.50	−29907.2		Not allowed
	M2: positive selection	ω_0_ = 0.15, ω_1_ = 1.00, ω_2_ = 5.39 *p*_0_ = 0.47, *p*_1_ = 0.50, *p*_2_ = 0.03	−29884.7	M2 vs. M1 2ΔLnL = 45 df = 2, *p* = 0.00	68, 116, **311**, 500
	M3: discrete	ω_0_ = 0.04, ω_1_ = 0.33, ω_2_ = 1.02 *p*_0_ = 0.26, *p*_1_ = 0.52, *p*_2_ = 0.22	−29726.0	M3 vs. M0 2ΔLnL = 1614 df = 4, *p* = 0.00	1, **20**, 46, **63**, **68**, **73**, **89**, **91**, 92, **96**, **97**, **116**, 139, **161**, **176**, 190, 204, 208, 212, **223**, **238**, 241, 263, **267**, **286**, **287**, 299, **306**, **311**, 319, **333**, **338**, **373**, **405**, **413**, **470**, **481**, **483**, **500**, 522, **531**, **534**, **544**, 593, 597, 660
	M7: β	*p* = 0.66, *q* = 1.06	−29714.7		Not allowed
	M8: β + ωS > 1	*p* = 0.71, *q* = 1.24 ω = 2.80 *p*_0_ = 0.96, *p*_1_ = 0.04	−29694.4	M8 vs. M7 2ΔLnL = 40.6 df = 2, *p* = 0.00	68, 116, **311**
Datamonkey^c^	SLAC				68, 129, 534
	FEL				68, 96, 129, 152, 208, 347, 371, 405, 463, 529, 534, 544, 569, 597, 639, 655, 789, 844
	REL				702, 789 823, 834, 844, 853

a *Codons identified by more than one maximum likelihood method are underlined*.

b *Only positively selected sites with Bayesian posterior probabilities equal or >95% are indicated. Sites with a posterior probability > 99% are highlighted in bold*.

c *Only positively selected sites with p < 0.01 (SLAC and FEL) or Bayes factor > 50 (REL) are shown*.

### Tissue Distribution of *tlr21, tlr22*, and *tlr23* Paralogues in *B. pectinirostris*

**Figure 8:**
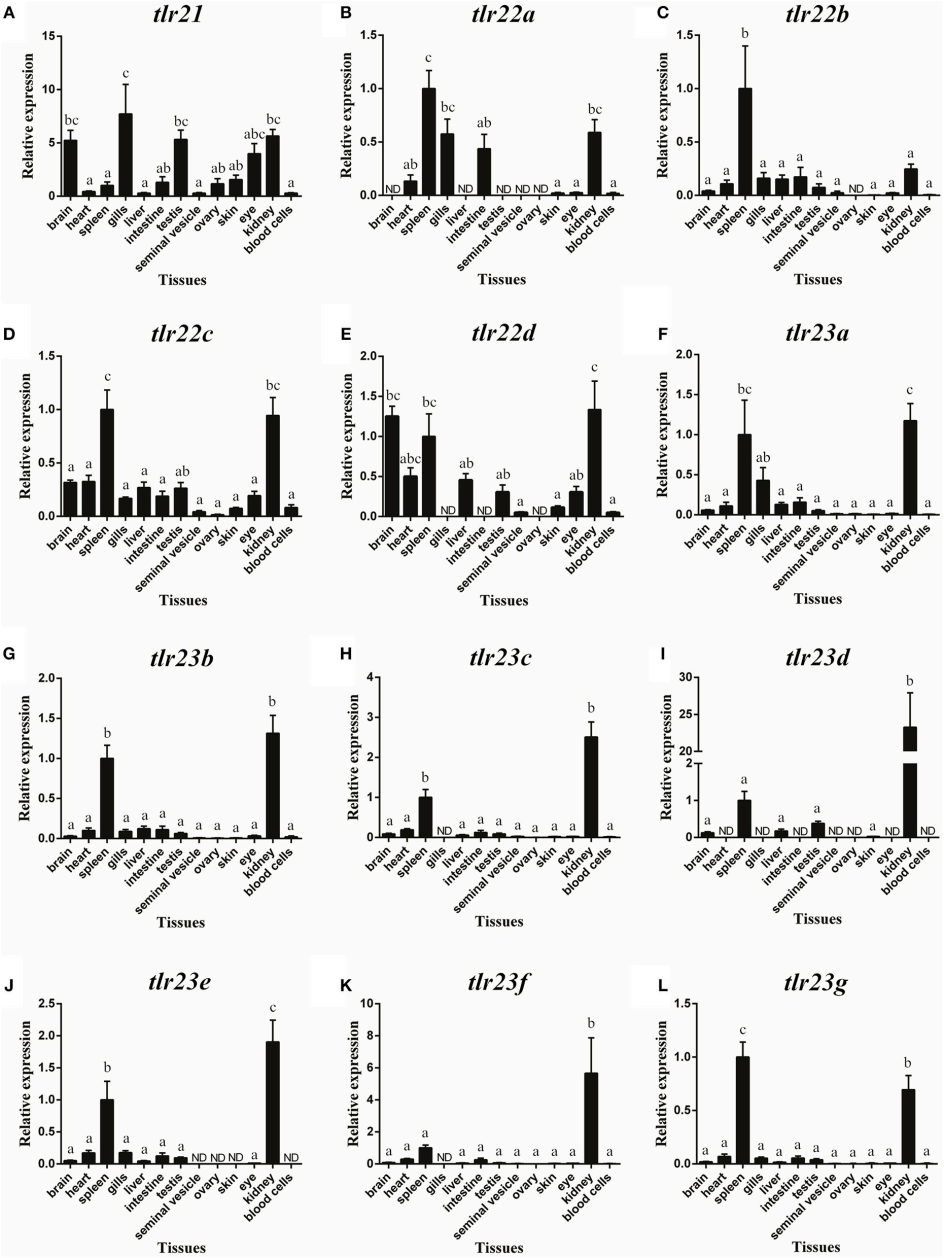
Tissue distribution of *tlr21* **(A)**, *tlr22* **(B-E)**, and *tlr23* **(F-L)** paralogues in *B. pectinirostris*. The levels of the respective mRNAs were determined using qPCR and normalized to the internal housekeeping gene *eef1*α. The results were expressed as mean ± SEM (*n* = 7). Columns with different letters are significantly differences with each other (*p* < 0.05, One-way ANOVA followed by Tukey's test).

*Tlr21, tlr22*, and *tlr23* paralogues showed distinguishable tissue expression patterns (Figure 8). They were all expressed in immune-related organs, i.e., spleen and kidney. In addition, *tlr21* was widely distributed and predominantly expressed in the brain, testis and eye (Figure 8A). Among *tlr22* paralogues (Figures 8B–E), only *tlr22c* was detectable in all tissues examined (Figure 8D). Compared with *tlr21* and *tlr22, tlr23* paralogues were exclusively expressed in the spleen and kidney (Figures 8F–L). The expression of these *tlr* genes in the seminal vesicle, ovary and skin was weak.

### Expression of the *tlr21, tlr22*, and *tlr23* Paralogues in Response to LPS and Poly(I:C) Challenges

**Figure 9:**
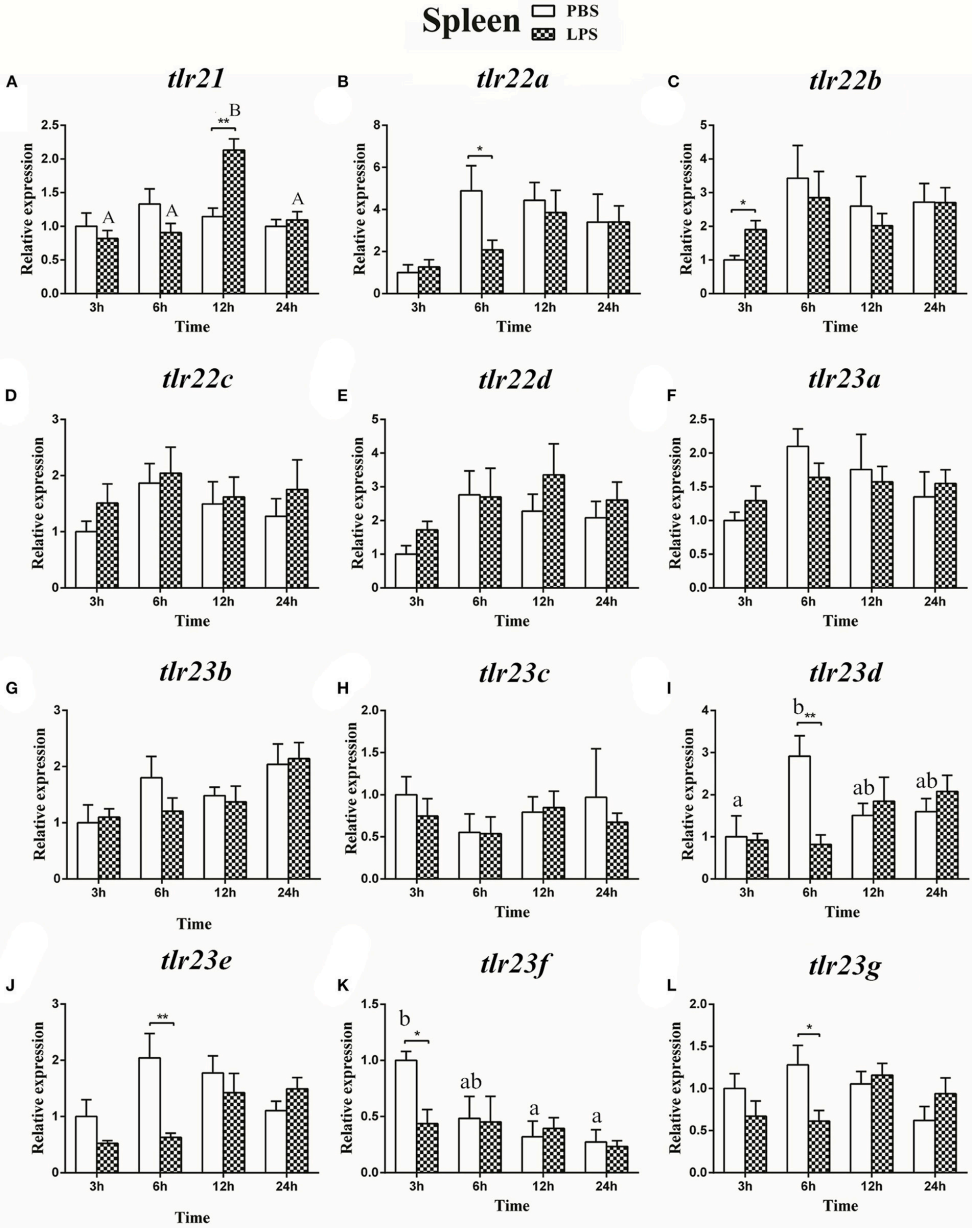
The relative expression of *tlr21* **(A)**, *tlr22* **(B-E)**, and *tlr23* **(F-L)** paralogues in the spleen after intraperitoneal injection with LPS. Relative expression of these 12 *tlr* genes in the kidney were examined at different time points (3, 6, 12, 24 h) by Real-time qPCR and all data were expressed as the mean ± SEM (*n* ≥ 5) and normalized to the expression of *eef1*α. Significant difference between PBS and LPS treated group was indicated with ^*^(*p* < 0.05, Student's *t*-test) or ^**^(*p* < 0.01, Student's *t*-test). Columns with different letters are significantly differences with each other (*p* < 0.05, One-way ANOVA followed by Tukey's test). Lowercase and uppercase letters indicate control and treated groups, respectively.

**Figure 10:**
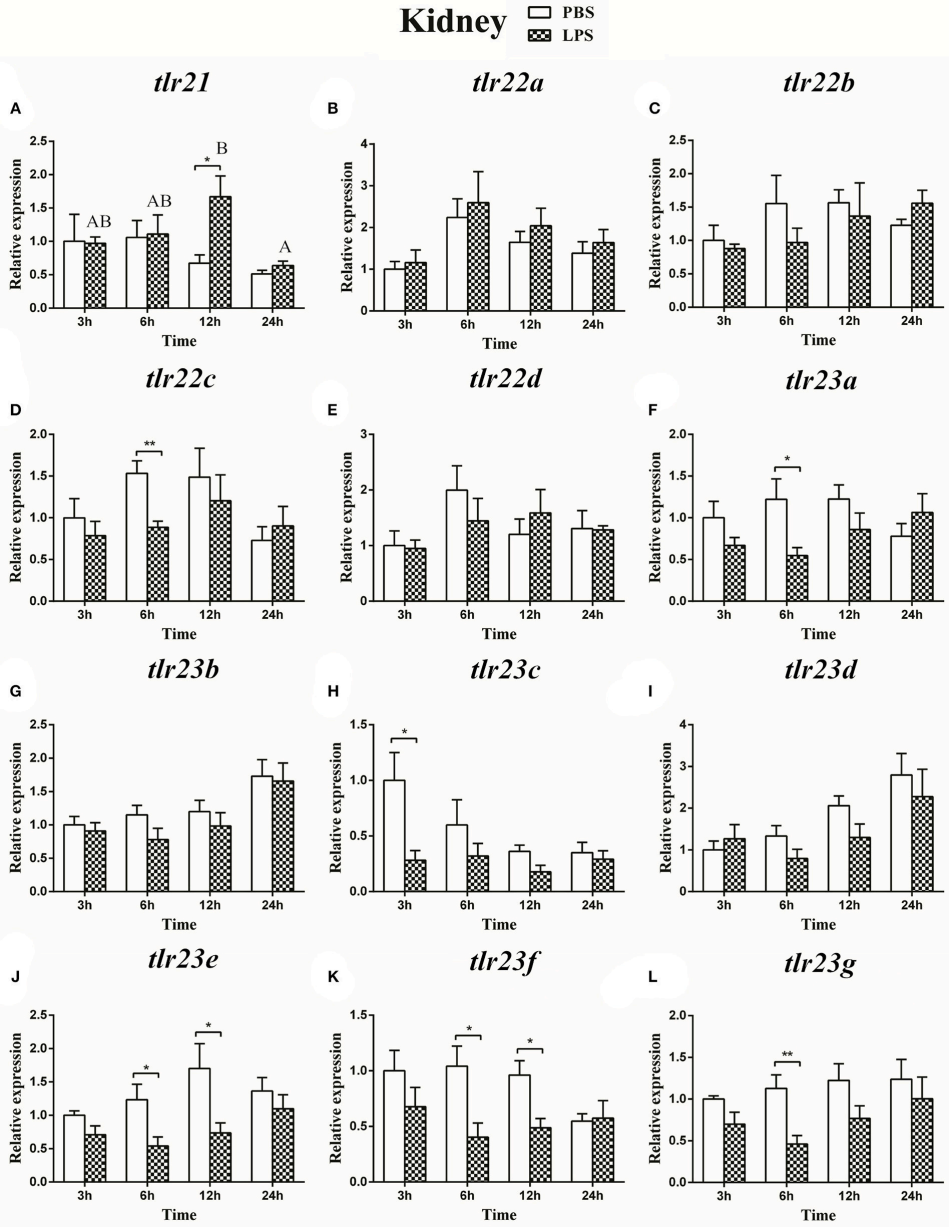
The relative expression of *tlr21* **(A)**, *tlr22* **(B-E)**, and *tlr23* **(F-L)** paralogues in the kidney after intraperitoneal injection with LPS. Relative expression of these 12 *tlr* genes in the kidney were examined at different time points (3, 6, 12, 24 h) by Real-time qPCR and all data were expressed as the mean ± SEM (*n* ≥ 5) and normalized to the expression of *eef1*α. Significant difference between PBS and LPS treated group was indicated with ^*^(*p* < 0.05, Student's *t*-test) or ^**^(*p* < 0.01, Student's *t*-test). Columns with different letters are significantly differences with each other (*p* < 0.05, One-way ANOVA followed by Tukey's test).

**Figure 11:**
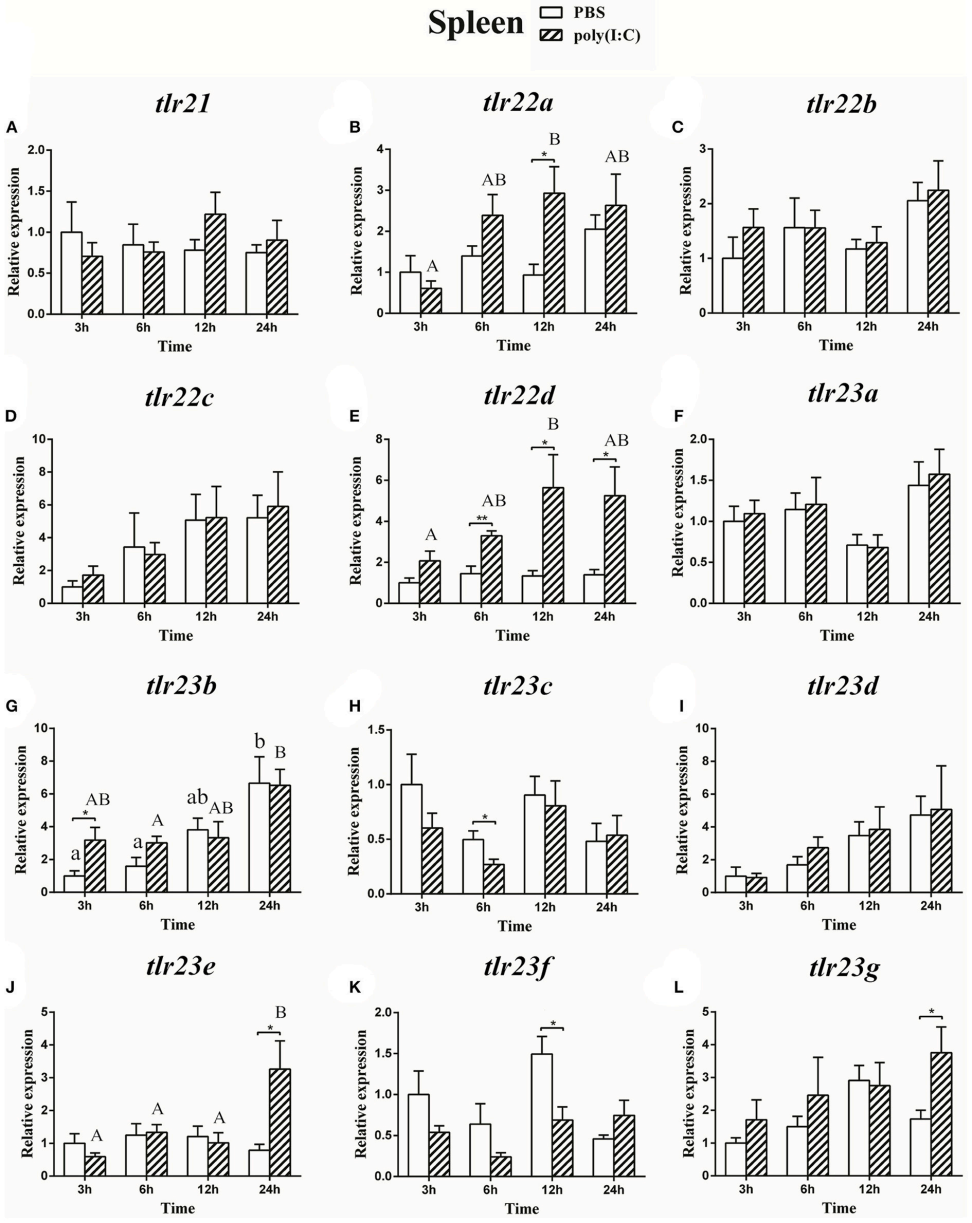
The relative expression of *tlr21* **(A)**, *tlr22* **(B-E)**, and *tlr23* **(F-L)** paralogues in the spleen after intraperitoneal injection with poly(I:C). Relative expression of these 12 *tlr* genes in the kidney were examined at different time points (3, 6, 12, 24 h) by Real-time qPCR and all data were expressed as the mean ± SEM (*n* ≥ 5) and normalized to the expression of *eef1*α. Significant difference between PBS and poly(I:C) treated group was indicated with ^*^(*p* < 0.05, Student's *t*-test) or ^**^(*p* < 0.01, Student's *t*-test). Columns with different letters are significantly differences with each other (*p* < 0.05, One-way ANOVA followed by Tukey's test). Lowercase and uppercase letters indicate control and treated groups, respectively.

**Figure 12:**
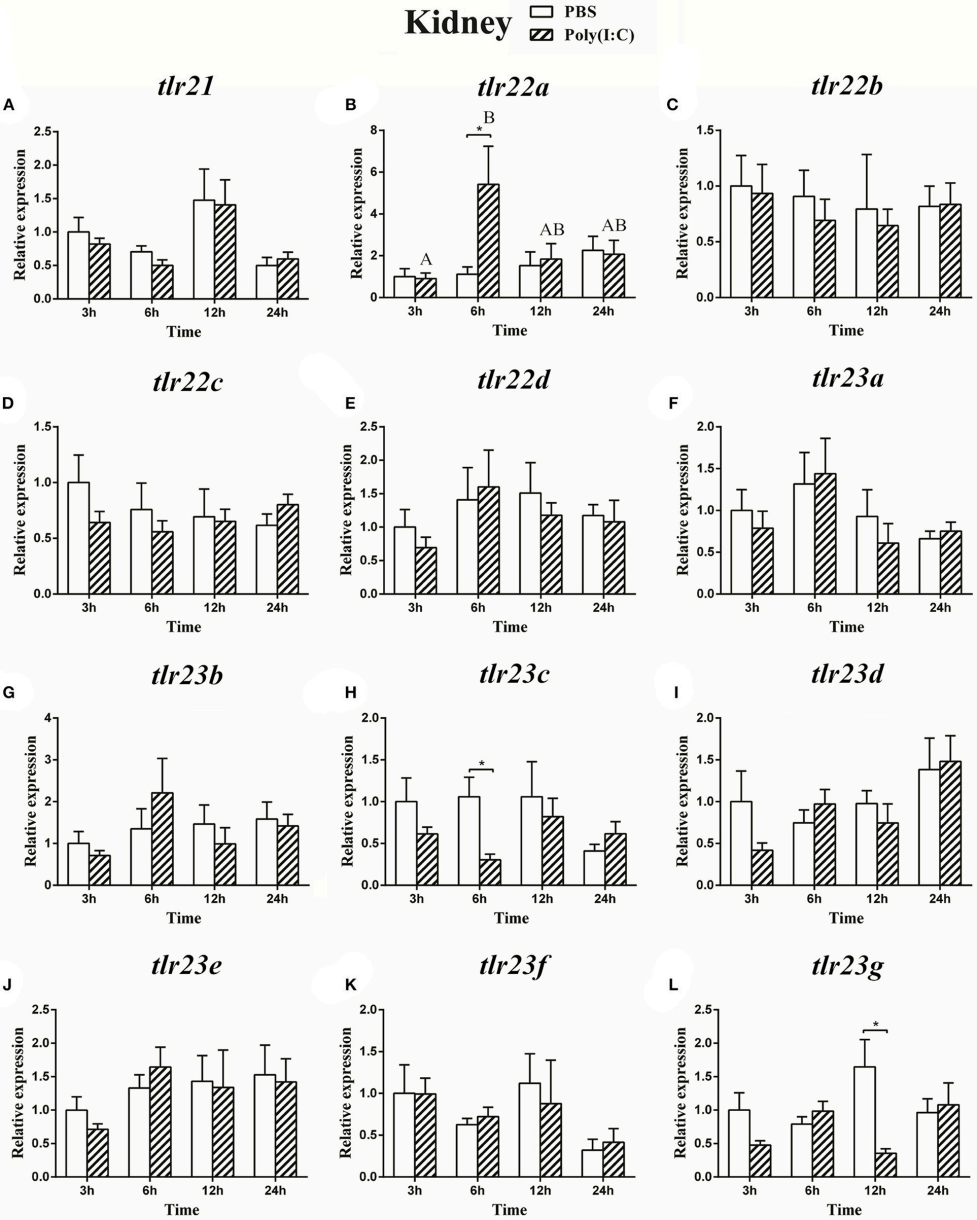
The relative expression of *tlr21* **(A)**, *tlr22* **(B-E)**, and *tlr23* **(F-L)** paralogues in the kidney after intraperitoneal injection with poly(I:C). Relative expression of these 12 *tlr* genes in the kidney were examined at different time points (3, 6, 12, 24 h) by Real-time qPCR and all data were expressed as the mean ± SEM (*n* ≥ 5) and normalized to the expression of *eef1*α. Significant difference between PBS and poly(I:C) treated group was indicated with ^*^(*p* < 0.05, Student's *t*-test). Columns with different letters are significantly differences with each other (*p* < 0.05, One-way ANOVA followed by Tukey's test).

The expression of *tlr21, tlr22*, and *tlr23* paralogues responsed differently to LPS (Figures 9, 10) and poly(I:C) (Figures 11, 12) challenges. The expression of *tlr21* was significantly up-regulated by LPS in the spleen and kidney at 12 hpi (Figures 9A, 10A). Among *tlr22* paralogues, LPS administration significantly stimulated *tlr22b* in the spleen at 3 hpi (Figure 9C), and down regulated *tlr22a* in the spleen at 6 hpi (Figure 9B) and *tlr22c* in the kidney at 6 hpi (Figure 10D). However, the expression profiles of these genes didn't show time-dependent significant differences. In response to poly(I:C) administration, *tlr22a* and *tlr22d* in the spleen showed clear significantly time-dependent increase pattern, and reached the significantly highest levels at 12 hpi (Figures 11B,E). In the kidney, only *tlr22a* significantly increased at 6 hpi after poly(I:C) stimulation, and followed by significantly dropping down for the rest experimental period (Figure 12B). Among *tlr23* paralogues, several genes were significantly down-regulated by LPS stimulation in both spleen and kidney (Figures 9, 10). However, the expression profiles of these genes didn't show time-dependent significant differences. Similar expression patterns were observed in response profiles of *tlr23c* and *tlr23g* after poly(I:C) stimulation in the kidney (Figures 12H,L). In spleen, except *tlr23c* and *tlr23f* showed similar down-regulation patterns (Figures 11H,K), *tlr23b, tlr23e*, and *tlr23g* were significantly stimulated by poly(I:C) at 3 or 12 hpi (Figures 11G,J,L). However, the expression profile of *tlr23b* showed significant increasing trend during sampling periods in both control and treated groups (Figure 11G). In contrast to *tlr23g* (Figure 11L), only *tlr23e* showed a clear significantly time-dependent increase pattern in the spleen, and reached the highest level at 24 hpi (Figure 11J).

## Discussion

Several types of gene duplication have been observed in genome to date, including whole genome duplication (43), segmental duplication (44), DNA-mediated duplicative transposition and retrotransposition (45). Paralogous or duplicated Tlr genes in teleosts, probably resulting from the third or fourth round of whole genome duplication event, have been identified in *Danio rerio* (46, 47), *Oncorhynchus mykiss* (48), and *Cyprinus carpio* (49). In the present study, we reported an extensive duplication of *tlr23* genes (7 paralogues). Previous study in Atlantic cod has reported an extensive duplication of *tlr22* genes (12 paralogues) (13). Such intensive duplication may be not only due to genome duplication but also due to other mechanisms of gene duplication. DNA-mediated duplicative transposition and retrotransposed duplication are ongoing process, continually expanding the genetic repertoire of modern organisms (45). A common result of DNA-mediated duplication is a new gene that preserves the intron-exon architecture and the cis-regulatory elements of the parental gene, whereas retrotransposed duplication often generates an intronless gene copy as a result of a process in which a spliced mRNA is reverse-transcribed into cDNA and spontaneously integrated into a random genomic location (45). Besides, it has been suggested that the influence of short tandem repeats may substantially increase the rate of duplication of a DNA segment located between them (50). In *B. pectinirostris*, both *tlr22* and *tlr23* paralogues showed similar intron-exon architectures. Moreover, short tandem repeat sequences were identified in the UTRs of *tlr22b, tlr22d, tlr23a*, and *tlr23d*. Thus, it is possible that *tlr22* and *tlr23* paralogues resulted from the DNA-mediated duplicative transposition.

Similar to other teleosts (13, 51, 52), there exists only one *tlr21* ortholog encoded by a single exon in *B. pectinirostris*. Besides, in *B. pectinirostris* and other fish species, Tlr21 molecules do not have an LRR-CT, in contrast to the chicken and goose TLR21 sequences (51). The structure of these molecules may suggest that the function of Tlr21 is highly conserved in teleosts. *B. pectinirostris tlr21* (Bp*tlr21*) was constitutively expressed at different levels in all examined tissues, which is similar to the expression patterns of *tlr21* in large yellow croaker (*L. crocea*) and Atlantic cod (13, 51). TLR21 in chicken (*G. gallus*) and zebrafish play a role in immune response to bacterial infection by recognizing CpG-oligodeoxynucletides (CpG-ODNs) as a danger signal (53, 54). In the present study, upon stimulation with LPS, the expression of Bp*tlr21* was significantly up-regulated in the spleen and kidney at 12 hpi in *B. pectinirostris*. In the mammal immune system, the complex of TLR4, CD14, and MD2 has been proved to be the receptor for LPS at the cell surface (55). Unlike in mammals, *tlr4* gene has been lost from the genomes of most fishes (9) including *B. pectinirostris*, and Tlr4 in fish does not recognize the stimulation of LPS (56). However, LPS does have multiple biological effects on fish including enhancing the production of immune related cytokines (57, 58). Recently, study in miiuy croaker (*Miichthys miiuy*) showed that NOD1 can identify LPS and activate the NF-κB signal pathway by recruiting RIPK2 and then promoting the expression of inflammatory cytokines to induce the resistance of organism against bacterial infection (59). Another study further demonstrated that scavenger receptor class B 2a (SRB2a), a novel isoform of the mammalian SRB2 gene, mediates LPS internalization for interaction with NOD1 and NOD2 to initiate NF-κB in teleost macrophages (60). The results from the present study suggest that BpTlr21 may be involved in immune response to bacterial infection in *B. pectinirostris*, and further studies should be focused on whether the stimulatory effects of LPS on Bp*tlr21* was mediated by SRB2a and NOD in *B. pectinirostris*.

The four *tlr22* orthologs cloned from *B. pectinirostris* grouped under a single clade in the phylogenetic tree, separately from the Atlantic cod orthologs of Bp*tlr22*, suggesting that the expansion of *tlr22* occurred independently during evolution of these two species. Bp*tlr22* orthologs are not adjacent in the *B. pectinirostris* genome. The genomic region surrounding *tlr22a* is conserved in comparison with *tlr22* from several fish species, and the region contains the genes *sh3kbp1, map3k15*, and *cnksr2*. It has been proposed that selection favors the organization of gene clusters to facilitate the coordinated control of gene expression and related biological processes (61). In mammals, Sh3kbp1 is involved in the B cell receptor signaling in normal lymphocytes (62). Besides, it has been reported that the B-cell response to CpG S-ODN is mediated through TLR9 (63). Therefore, conserved syntenic localization between Tlr22a and Sh3kbp1 suggests that Tlr22a may participate in biological processes related to B cells in teleosts. Map3k15 (ASK3) is likely a component of ASK1 (Map3k5) signalosome and can interact with ASK1 (64), and ROS-dependent activation of TRAF6-ASK1-p38 pathway is crucial for TLR4-mediated innate immunity (65), which suggest that Map3k15 may take part in Tlr22a signaling pathway. Further study would be interesting to examine these adjacent genes involved in the Tlr22a mediated signaling pathway in *B. pectinirostris*.

In other teleosts, poly(I:C) challenge up-regulated the expression of *tlr22* in many tissues (66, 67). It was reported, in tiger pufferfish, that Tlr22 localizes to the cell surface and recognizes long-sized dsRNA or poly(I:C) and links the IFN-inducing pathway via the TRIF adaptor (68). In the present study, the expression of *tlr22a* and *tlr22d* in spleen, and *tlr22a* in kidney showed clear time-dependent up-regulation after poly(I:C) stimulation. These results may suggest that the function of Tlr22a and Tlr22d might play a role in innate immune response to virus. Nevertheless, some Bp*tlr22* paralogues (including *tlr22a*) showed a response after LPS stimulation. A study in Atlantic cod indicated that most of *tlr22* orthologs transcripts are up-regulated after bacterial bath challenge (13). However, it is worth noting that, after LPS stimulation, the expression profiles of Bp*tlr22* paralogues didn't show time-dependent significant differences. Therefore, further studies would be necessary to investigate the possibility that the expansion of Tlr22 likely increases the detectable ligand repertoire, e.g., to recognize dsRNA and PAMPs from pathogen origin.

The function of TLR23 is still largely unknown (13). *B. pectinirostris* encoded 7 copies of *tlr23* genes and possessed the largest group of Tlr23 in vertebrates sequenced. It is noteworthy that all BpTlr23 except Tlr23a clustered under a single clade in phylogenetic tree, which suggests that BpTlr23 under the same clade may evolve independently in comparison with BpTlr23a and other teleost Tlr23. We found that *Tlr23a* genes in several fish species are often adjacent to the *ppme1-like* gene, which suggests that the function of *tlr23a* may be conserved in different teleost species. Recently, it was found that activation of TLR4 signaling pathway will increase the expression of MFHAS1, which further inhibits expression of inflammatory factors and plays a role in negatively regulating TLR4 signaling pathway (69). During this process, MFHAS1 combines with the B and C subunits of PP2A, which involves up-regulation of PPME-1 (70). Therefore, it is possible that *ppme1* may be involved in the negative regulation of signaling pathway of *tlr23a*, and even other *tlr23* paralogues.

*Tlr23* paralogues in *B. pectinirostris* responded differently to LPS and poly(I:C) challenges. *Tlr23e* and *tlr23g* were significantly up-regulated in the spleen upon poly(I:C) stimulation, which suggest that *tlr23e* and *tlr23g* may participate in antiviral immune processes. In contrast to up-regulation, several *B. pectinirostris tlr23* paralogues showed down-regulation after LPS and poly(I:C) challenges. Atlantic cod *tlr23a* is also significantly reduced upon a bath challenge with Gram-negative bacteria *V. anguillarum* (13). In teleost fish, several studies have identified subsets of microRNAs (miRNAs) that are differentially expressed in organs challenged with DNA or RNA virus, LPS or poly (I:C) (71). Based on the use of bioinformatics approaches and whole transcriptome analysis, increasing studies have discovered that miRNAs negatively regulate the expression of Tlr genes (72). It would be necessary to predict miRNA regulators of *tlr23* paralogues and elucidate their roles in the regulation of *tlr23* paralogues in *B. pectinirostris*.

The average number of synonymous changes was higher than the non-synonymous substitutions in all pairwise comparisons between *tlr11* genes in *B. pectinirostris*, indicating overall purifying selection, likely due to functional constraints (73). In fish, the prevalence of purifying selection signatures has been reported not only in *tlr* genes (13) but also in other immune-related genes, such as antimicrobial peptides (39). Nevertheless, substitution rates were not uniform across *tlr11* paralogues in *B. pectinirostris* and the observed differences contribute to explaining how the TIR domains of Tlr21, Tlr22, and Tlr23 are more conserved than their LRR regions. The TIR domain is generally conserved across species as well as between different TLRs, since it is involved in signal transduction (74).

Homology modeling of *B. pectinirostris* Tlr23a based on human TLR5 revealed a characteristic horseshoe-shaped structure with a single ectodomain architecture. Most positively selected sites were found in the ectodomain, especially in the convex side of the extracellular solenoid structure, which is most important for ligand binding. Non-synonymous substitutions at the positively selected sites may affect ligand specificity through changes in the amino acids within the beta sheets or in the convex surface of the horseshoe-shaped domain (75). For example, variations in the LRR coil at position 68 between small polar (S and T), positively charged (R, K and H) and large hydrophobic aromatic (F and W) amino acids will likely affect the polarity and structure of the ectodomain, thus affecting ligand specificity. This functional diversification of the *B. pectinirostris* Tlr11 family through positive selection may be linked to adaptation to evolving pathogens.

In conclusion, we identified and annotated 12 *tlr* genes (one *tlr21*, 4 *tlr22*, and 7 *tlr23*) representing all members of the high expanded Tlr11 family in the mudskipper *B. pectinirostris*. The expanded Tlr11 family in *B. pectinirostris* provides a good model to better understand how and why so many TLR genes have been retained during vertebrate evolution.

## Author Contributions

HQ was involved in entire study. JF analyzed the positive selection. XY, HW, and HY performed the partial synteny and chromosome location analyses. YZ, SH, DL, and QW analyzed results. SC and WH conceived and supervised the project, analyzed results and prepared the manuscript.

### Conflict of Interest Statement

The authors declare that the research was conducted in the absence of any commercial or financial relationships that could be construed as a potential conflict of interest.

## Abbreviations

Bp: *Boleophthalmus pectinirostris*
CD: cluster of differentiation
CDS: complete codinig sequence
CpG-ODNs: CpG-oligodeoxynucletides
CT: C-terminus
MD-2: myeloid differentiation factor 2
*eef1*α: *eukaryotic translation elongation factor 1*α
hpi: hours post injection
LPS: lipopolysaccharide
LRRs: leucine-rich repeats
Map3k: mitogen-activated protein kinase kinase kinase
miRNA: microRNA
NF-κB: nuclear factor-κB
NOD: nucleotide-binding oligomerization domain
NT: N-terminus
PAMPs: pathogen-associated molecular patterns
poly(I:C): polyinosinic-polycytidilic acid
*ppme1*: *protein phosphatase methylesterase-1*
RIPK2: receptor-interacting serine-threonine kinase 2
SEM: standard error of the mean
Sh3kbp1: SH3-domain kinase binding protein 1
SRB: scavenger receptor class B
TIR: Toll-Interleukin-1 receptor
TLR: Toll-like receptor
TRIF: TIR-domain-containing adapter-inducing interferon-β
UTR: untranslated region.
