# Supplementary material for: Paralogues From the Expanded Tlr11 Gene Family in Mudskipper (Boleophthalmus pectinirostris) Are Under Positive Selection and Respond Differently to LPS/Poly(I:C) Challenge
Source: Front Immunol. 2019 Feb 28;10:343. doi: 10.3389/fimmu.2019.00343 (PMC6403153; doi:10.3389/fimmu.2019.00343)
Supplement: Supplementary file 1 [file Data_Sheet_1.PDF]

Supplementary table 1

The primers for amplification of open reading frames of *tlr21*, *tlr22* and *tlr23* paralogues in this study.

Primer	Sequence(5'-3')
tlr21 F	GTCTGAAGAGAGTGATGACAAGTC
tlr21 R	GATAAATTTCAATAAATGTTTATAC
tlr22a F	ATGGGTCATGAATACCAAAAATCAC
tlr22a R	TCATTCTTCGTCTGTTACAGTGAGC
tlr22b F	AAGTGGTCACTGTTCAGTCTGTAC
tlr22b R	GATGGTTTTCCTGCTCCTGTAAAC
tlr22c F	ATGGACTCTACAATCATGAGATCATTTG
tlr22c R	TTAGTCAATAAGGTGAACACTGTCTC
tlr22d F	GTGGCACTTCCTCAAGAAATGAAG
tlr22d R	TATTGTCACTGAAAGCAGTCTCAG
tlr23a F	TAAACCTACAGCGTCGCCATGTC
tlr23a R	ATTCCTCCTCTGATTGGACACTG
tlr23b F	ATGGACCCTCACACGTCTCCTCTCGTC
tlr23b R	GACCGTCATCTGGACTTCAGCAG
tlr23c F	GAGCATGAGCCGTCACAGCTGTTC
tlr23c R	CAACAGTGACCAGGTTGCAGTTAAAC
tlr23d F	CTCAGGATGAGGAGTCAGAGCAC
tlr23d R	CTAATAAACATTAGCGGTCAGTGG
tlr23e F	CACAGGAACTTTCTTCAAAATGT
tlr23e R	CTACACATTAGCAGCATGACGGTTG
tlr23f F	CAGGATGTGGCCTGCGAGCACTTC
tlr23f R	ACTTTTACGGTGACCAGGTTGCAG
tlr23g F	TCAGATTTGTCCGCTTTAACTAC
tlr23g R	GTTTTAGGTGCTAAATGTTAGTAG

[a] F indicates forward; R, reverse.

Supplementary table 2

The primers for amplification of the 5' and 3' ends of cDNAs of *tlr21*, *tlr22* and *tlr23* paralogues.

Primer	Sequence(5'-3')
tlr21 5'-GSP Outer	TGGTAAATGAACAAAGCTCTTGTC
tlr21 5'-GSP Inner	ATGCAGTTGTTAAAGCTGTAACTG
tlr21 3'-GSP Outer	AACTCTGTCTACATCACAGAGAC
tlr21 3'-GSP Inner	GACGTAACATTGTCGATAACATTG
tlr22a 5'-GSP Outer	TCACTGTTGGAGGAATATCTGTAG
tlr22a 5'-GSP Inner	CTGTGCGAGATTACCACTGTCAC
tlr22a 3'-GSP Outer	GTCATATAACGTCCATGATGAAG
tlr22a 3'-GSP Inner	GACGAGAGGAAGGACGTGCTGATC
tlr22b 5'-GSP Outer	ATGTTGTTGGAGCTCAGGTCGATG
tlr22b 5'-GSP Inner	CCGTCCAACAGGTAACAGTTCTG
tlr22b 3'-GSP Outer	TTCAGCAGATTTCACCTGAGTTAC
tlr22b 3'-GSP Inner	CAGAAGCGAGCGGAGAGTCTGTAC
tlr22c 5'-GSP Outer	TGAAATGTTGTTGTTCCTCAGATC
tlr22c 5'-GSP Inner	GGGACCTGTGTCAGTTGGTTCTG
tlr22c 3'-GSP Outer	TGGCTTTATCTGTTTCATATCCAG
tlr22c 3'-GSP Inner	ACCTACGCCTACTACATCTTCTTG
tlr22d 5'-GSP Outer	TTTGGTGCTACTGTTTGAATACG
tlr22d 5'-GSP Inner	GGTGAGTTTCTGTTTGGAGCAATC
tlr22d 3'-GSP Outer	GCGTAGAGGATCAGTACGATG
tlr22d 3'-GSP Inner	TCAGCTCACCTGTCTCCTTATTAC
tlr23a 5'-GSP Outer	TTGGTCAGAGAAATCTGCTGCATG
tlr23a 5'-GSP Inner	GCCGTGATGTCTGCTAAGTTACTG
tlr23a 3'-GSP Outer	GTCTCAGCACAGAGCCGTGTTCTG
tlr23a 3'-GSP Inner	CACCACAGTGTCCAATCAGAGGAG
tlr23b 5'-GSP Outer	GTGAGGTTAAAGTTCCTGACGCTG
tlr23b 5'-GSP Inner	GGGTCCAGACTGCAGCTGCGTAG
tlr23b 3'-GSP Outer	CGTTCGTTTCCTACAACGTTCACG
tlr23b 3'-GSP Inner	CATCACAGACGCGATTTACAGCAG
tlr23c 5'-GSP Outer	GAGAGCTGTCTGTTTGATGCTCTG
tlr23c 5'-GSP Inner	GACTGTCAGTTATTCTCACAGCTG
tlr23c 3'-GSP Outer	AGCAAGTTCACTCCAGAGAGATAC
tlr23c 3'-GSP Inner	CATCATTGAGAACATCACAGATG
tlr23d 5'-GSP Outer	ACAACATAATCTCAGTTACAGCAG
tlr23d 5'-GSP Inner	TCCTTCTTCAATGTGTGAGATGTC
tlr23d 3'-GSP Outer	CGTCTTCAATTTATCAACTGTGAC
tlr23d 3'-GSP Inner	CAATTCACTCACACATCTAAGCTG

tlr23e 5'-GSP Outer	CCGTCTTCTATGAGTGAAATGTG
tlr23e 5'-GSP Inner	CAGATCTGTCTGTTTGATTGTCTC
tlr23e 3'-GSP Outer	TGTGTCCTACAACTGTCACGATG
tlr23e 3'-GSP Inner	GGAAACCGATCATTGAGAACATC
tlr23f 5'-GSP Outer	TTGTCCAAGAAATAATGCCTCAAG
tlr23f 5'-GSP Inner	TCTGACTCTCTAATTGTTACAATG
tlr23f 3'-GSP Outer	AAGTTCAAGTGGTCCGAGCATATC
tlr23f 3'-GSP Inner	GATGGAGACTGTGTCTGCACCAC
tlr23g 5'-GSP Outer	TGGACACTGTGAAACTATGTTGAC
tlr23g 5'-GSP Inner	GTAGCTTTCCTAATGGCTTGTAAG
tlr23g 3'-GSP Outer	CTGGAGGAGATCTCGTCCAATCAG
tlr23g 3'-GSP Inner	TGAAGAGGTGCACGTATTTGAGC

Supplementary Table 3
The characteristics of tandem repeats in *tlr22b*, *tlr23a* and *tlr23d*.

Gene	conserved tandem repeative sequence	length of each tandem repeat (bp)	copies of tandem repeat	total length of tandem repeats (bp)	position of tandem repeats in cDNA sequence (5'-3', bp)	region of tandem repeats
tlr22b	AGAGGACA(G/C)AGAGGACAGAGGACAGAGAGGAC	32	3	96	9-104	5'-UTR
	TTAGTC(C/T)TGGT	11	50	549	2837-3385	mainly within 3'-UTR
tlr23a	AGAAGAACATC	11	11	121	1-121	5'-UTR
	CGTCCAATCAGAGGAGGAGTAACACCGCAG	30	5	150	2987-3136	mainly within 3'-UTR
tlr23d	AT(A/G)CAGTAAAGTACTGCAGTAATA(C/T)AG	27	9	243	3714-3956	3'-UTR

Supplementary Table 4

The details about the chromosome location of *tlr21*, *tlr22* and *tlr23* paralogues.

Chr: chromosome, rel_loc: relative location.

BpTlr11 family	chr	alignment_length (bp)	query_start (bp)	query_end (bp)	chr_start (bp)	chr_end (bp)	chr_length (bp)	rel_loc
tlr21	Chr7	3497	1	3497	13357551	13361047	47326034	0.282245307
tlr22a	Chr12	2546	22	2567	20284389	20281844	37111425	0.546580709
tlr22b	Chr8	2505	132	2636	3164798	3162294	44440466	0.07121433
tlr22c	Chr7	2526	44	2569	8150812	8148287	47326034	0.172226813
tlr22d	Chr17	4021	2	4022	37326629	37322661	43774098	0.852710409
tlr23a	Chr6	2483	172	2654	19660101	19662583	41979960	0.468321099
tlr23c	Chr11	2550	19	2568	23185608	23183059	38348280	0.604606204
tlr23d	Chr11	2401	1	2401	35058319	35055919	38348280	0.914208382
tlr23e	Chr12	3827	1	3827	23436309	23432487	37111425	0.631511967
tlr23f	Chr11	1567	924	2490	23175758	23174192	38348280	0.604349348
tlr23g	Chr18	2470	23	2492	22604352	22601884	41117269	0.549753244

Supplementary Table 5. Codon based Z-test of positive selection within *tlr11* paralogues. The modified Nei-Gojobori method with Jukes-Cantor correction and an assumed transition/transversion bias = 2 was used in the analysis. The test statistic (dN-dS) and the probability of rejecting the null hypothesis of strict-neutrality (dN = dS) in favour of the alternative hypothesis (dN > dS) are shown above and below the diagonal, respectively. Significance was set at P<0.05. A total of 793 codons were included in the final dataset, since all positions containing gaps and missing data were eliminated.

tlr23d		0.624	5.877	4.497	5.064	5.441	4.296	2.959	2.002	4.395	5.440	2.728
tlr21	1.000		3.435	1.934	0.728	0.411	0.490	2.327	1.933	2.608	0.892	2.889
tlr23b	1.000	1.000		3.238	6.167	6.122	2.875	3.753	6.117	5.073	6.040	3.971
tlr23a	1.000	1.000	1.000		4.738	5.196	1.522	4.343	4.917	5.369	4.307	3.768
tlr23g	1.000	1.000	1.000	1.000		3.631	3.752	3.912	3.372	3.911	5.749	2.704
tlr23f	1.000	1.000	1.000	1.000	1.000		3.949	3.795	3.120	4.421	5.846	3.122
tlr22b	1.000	1.000	1.000	1.000	1.000	1.000		7.003	4.922	7.694	2.739	7.611
tlr22a	1.000	1.000	1.000	1.000	1.000	1.000	1.000		3.539	7.108	3.355	7.081
tlr23c	1.000	1.000	1.000	1.000	1.000	1.000	1.000	1.000		5.315	4.420	3.602
tlr22d	1.000	1.000	1.000	1.000	1.000	1.000	1.000	1.000	1.000		3.171	7.093
tlr23e	1.000	1.000	1.000	1.000	1.000	1.000	1.000	1.000	1.000	1.000		2.713
tlr22c	1.000	1.000	1.000	1.000	1.000	1.000	1.000	1.000	1.000	1.000	1.000	

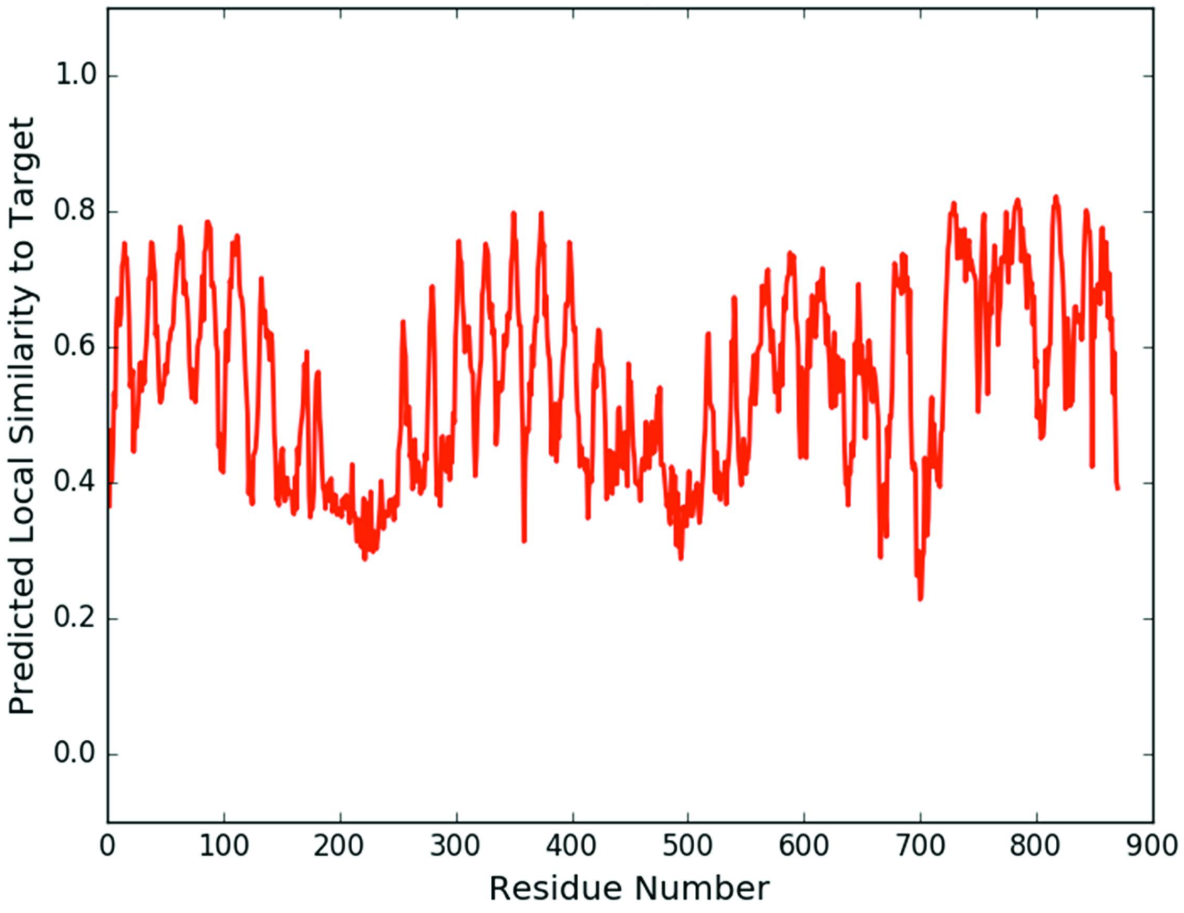

Supplementary Fig. 1. Local quality plot showing, for each residue of the model on the x-axis, the expected similarity to the native structure (y-axis). Typically, residues showing a score above 0.6 are expected to be of high quality.

Tandem repeats in 5'-UTR of *tlr22d*

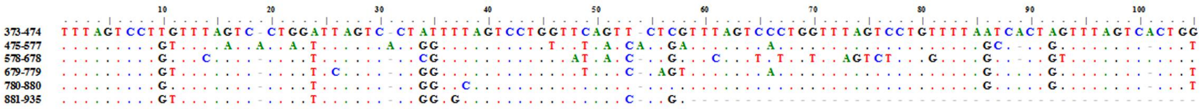

Supplementary Fig. 2. Conserved tandem repeat sequences in *tlr22d*. DNA sequence identities to the top sequence are indicated by a same color dot. The left number shows the position of the repeat sequences in cDNA sequence.

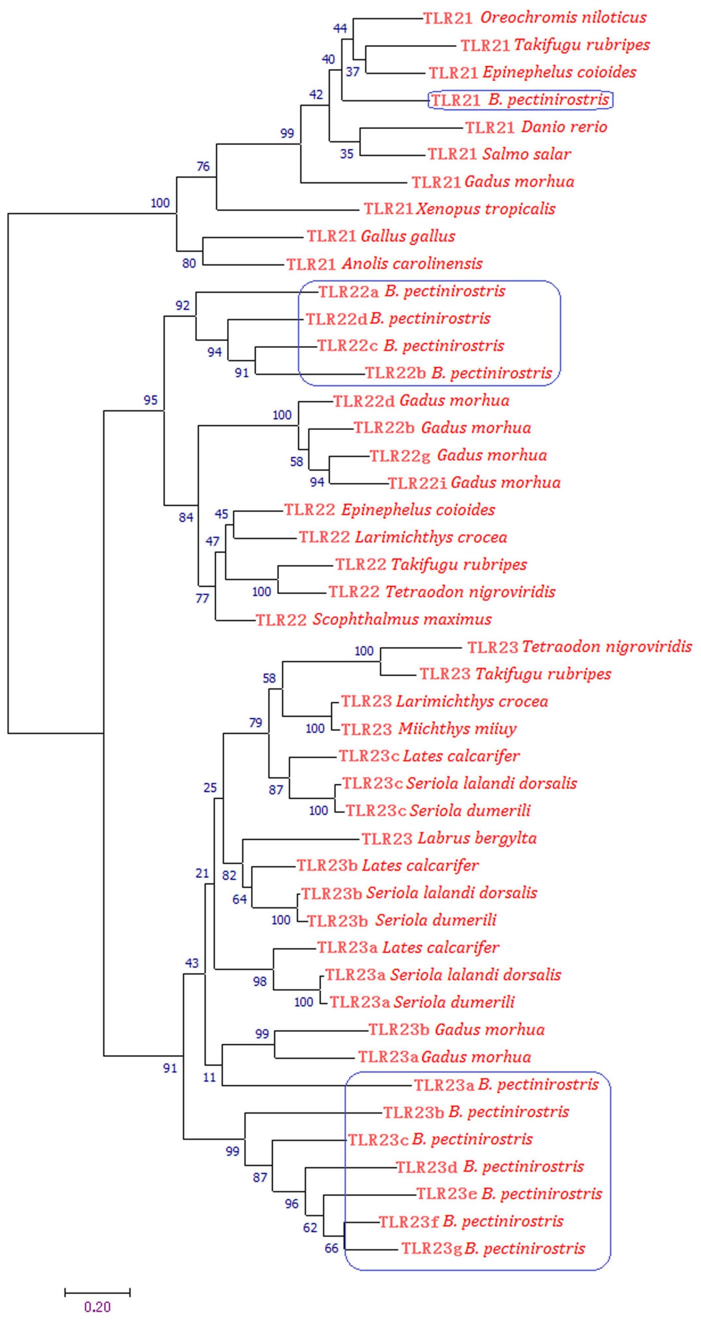

Supplementary Fig. 3. Phylogenetic analysis of TLR21, TLR22 and TLR23 using MEGA 7 by the Maximum Likelihood method and 1000 replications of bootstrap. Proteins of *B. pectinirostris* are highlighted within blue box.

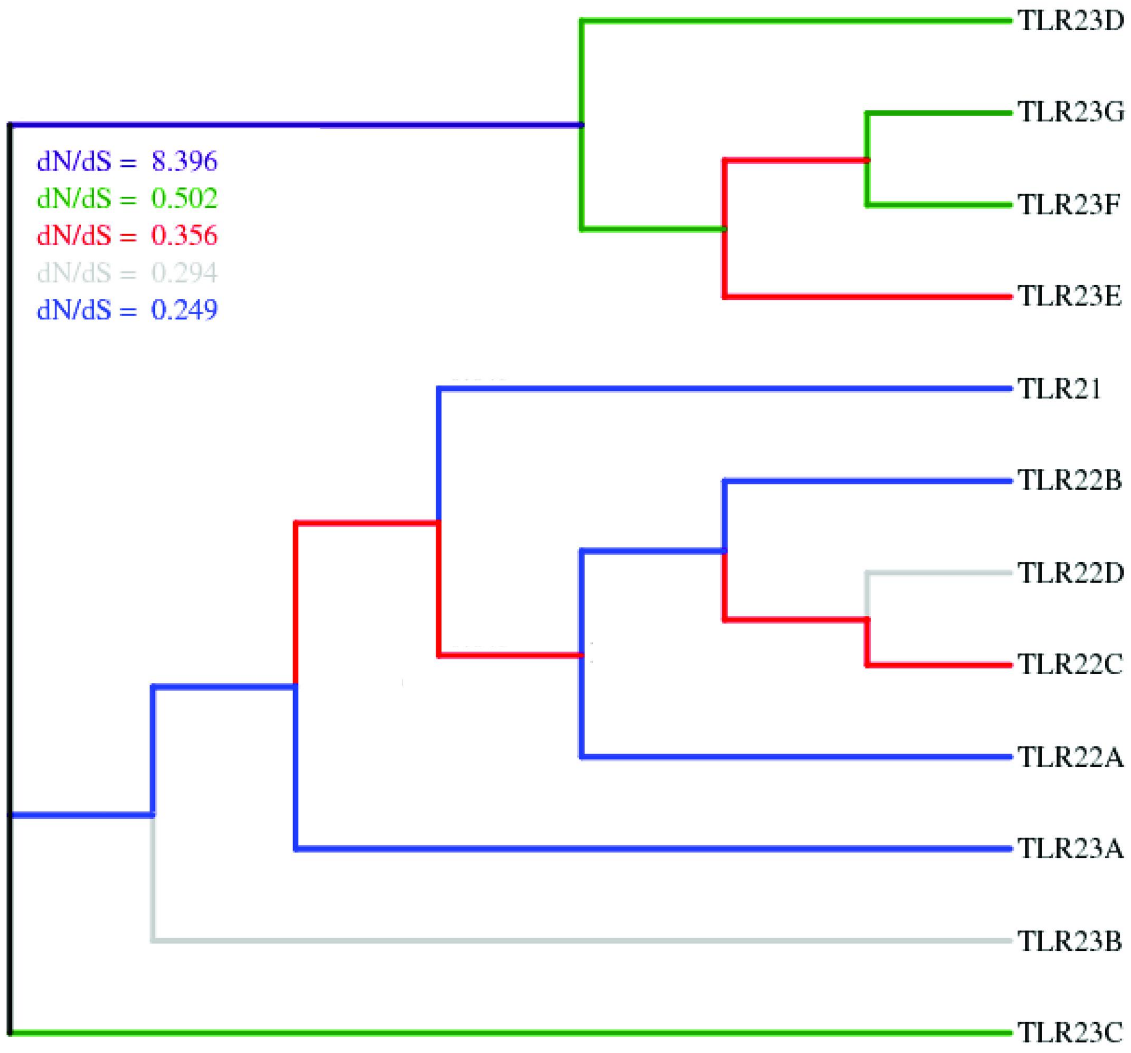

Supplementary Fig. 4. Unscaled GA-branch tree with the best c-AIC branch partitioning. Branches are colored according to the dN/dS ratios of the 5 rate classes in the model and the corresponding values are also indicated.

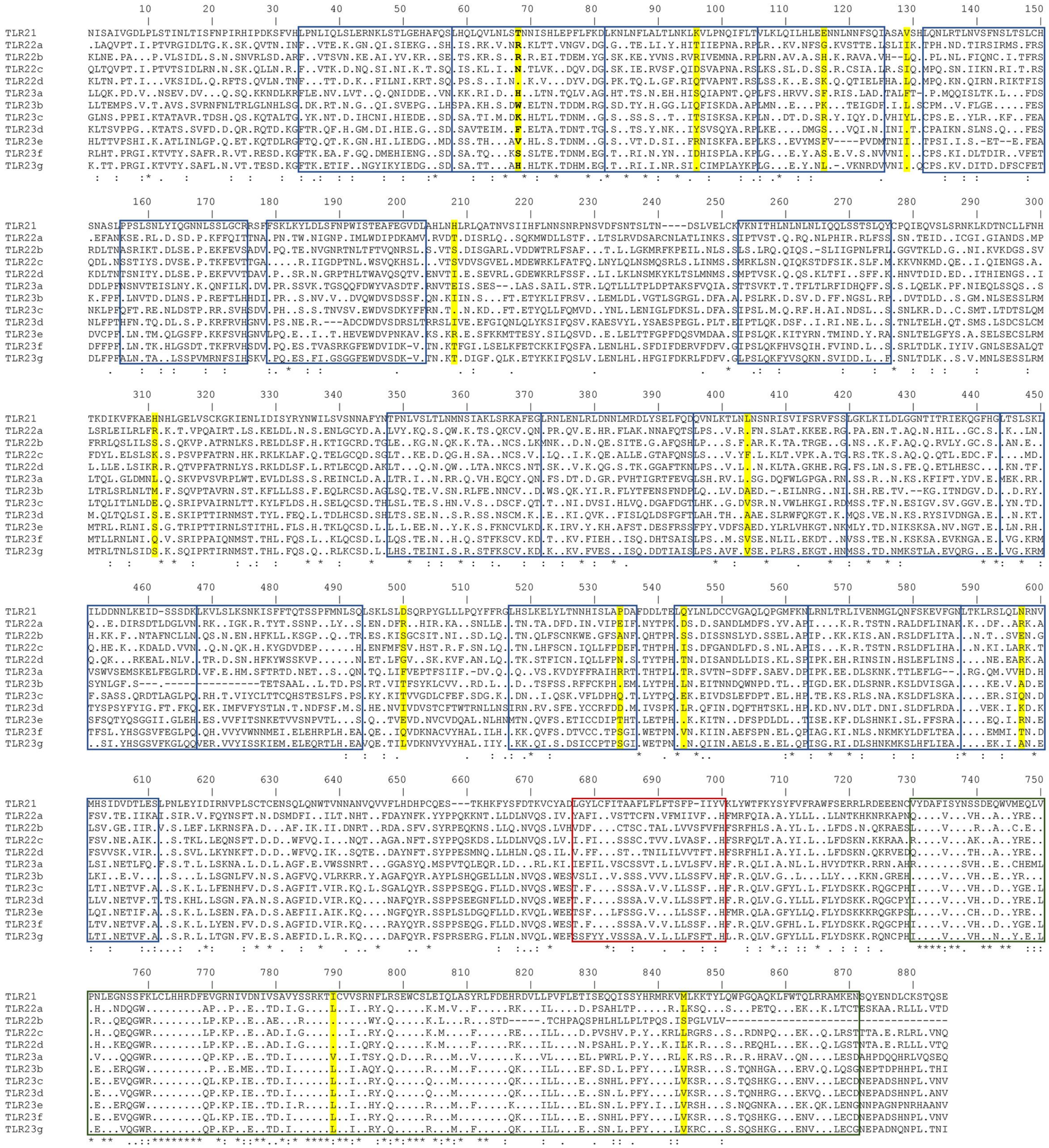

Supplementary Fig. 5. Positively selected sites on the multiple sequence alignment of *B. pectinirostris* Tlr21, Tlr22 and Tlr23. Amino acid residues identical to Tlr21 are represented by a dot and alignment gaps are indicated by a dash. Conserved substitutions and identical residues between sequences are designated by a colon and an asterisk, respectively. Positively selected

sites identified by more than one likelihood model are highlighted in yellow. LRR regions, transmembrane and TIR domains are boxed in blue, red and green, respectively. Position 68 is in bold to indicate that this codon was found to be under positive selection by all models tested except REL.
